# Cross-Neutralization of a SARS-CoV-2 Antibody to a Functionally Conserved Site Is Mediated by Avidity

**DOI:** 10.1016/j.immuni.2020.10.023

**Published:** 2020-12-15

**Authors:** Hejun Liu, Nicholas C. Wu, Meng Yuan, Sandhya Bangaru, Jonathan L. Torres, Tom G. Caniels, Jelle van Schooten, Xueyong Zhu, Chang-Chun D. Lee, Philip J.M. Brouwer, Marit J. van Gils, Rogier W. Sanders, Andrew B. Ward, Ian A. Wilson

**Affiliations:** 1 Department of Integrative Structural and Computational Biology, The Scripps Research Institute, La Jolla, CA 92037, USA; 2 Department of Medical Microbiology, Amsterdam UMC, University of Amsterdam, Amsterdam, the Netherlands; 3 Department of Microbiology and Immunology, Weill Medical College of Cornell University, New York, NY 10021, USA; 4 IAVI Neutralizing Antibody Center, The Scripps Research Institute, La Jolla, CA 92037, USA; 5 Consortium for HIV/AIDS Vaccine Development (CHAVD), The Scripps Research Institute, La Jolla, CA 92037, USA; 6 The Skaggs Institute for Chemical Biology, The Scripps Research Institute, La Jolla, CA 92037, USA

## Abstract

Most antibodies isolated from individuals with coronavirus disease 2019 (COVID-19) are specific to severe acute respiratory syndrome coronavirus 2 (SARS-CoV-2). However, COVA1-16 is a relatively rare antibody that also cross-neutralizes SARS-CoV. Here, we determined a crystal structure of the COVA1-16 antibody fragment (Fab) with the SARS-CoV-2 receptor-binding domain (RBD) and negative-stain electron microscopy reconstructions with the spike glycoprotein trimer to elucidate the structural basis of its cross-reactivity. COVA1-16 binds a highly conserved epitope on the SARS-CoV-2 RBD, mainly through a long complementarity-determining region (CDR) H3, and competes with the angiotensin-converting enzyme 2 (ACE2) receptor because of steric hindrance rather than epitope overlap. COVA1-16 binds to a flexible up conformation of the RBD on the spike and relies on antibody avidity for neutralization. These findings, along with the structural and functional rationale for epitope conservation, provide insights for development of more universal SARS-like coronavirus vaccines and therapies.

## Highlights

• X-ray and EM structures of cross-neutralizing antibody COVA1-16 with SARS-CoV-2 RBD
• COVA1-16 binding to SARS-CoV-2 RBD is dominated by CDR H3
• COVA1-16 binds to a highly conserved non-RBS epitope but still competes with ACE2
• IgG avidity is the key for the cross-neutralization activity of COVA1-16

## Graphical Abstract

**Figure:**
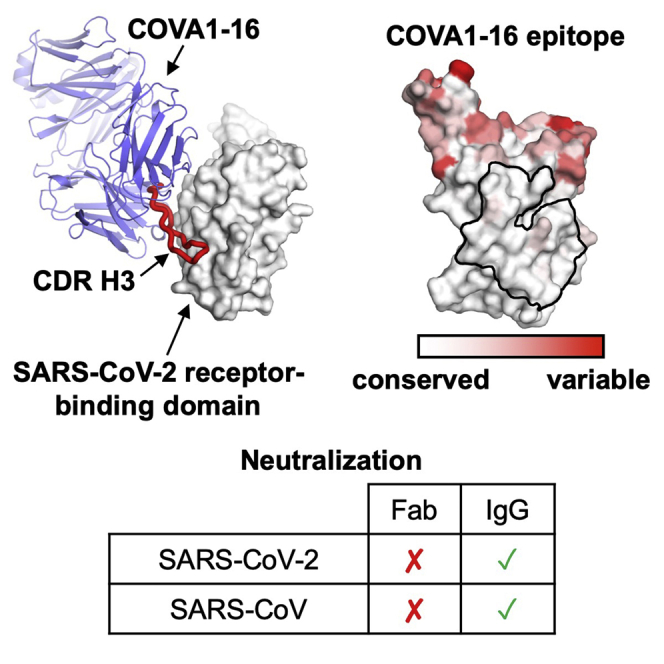

## Teaser

COVA1-16 is a SARS-CoV-2 antibody from an individual with COVID-19 that cross-neutralizes SARS-CoV. Liu et al. reveal that COVA1-16 binds to a highly conserved epitope using a long CDR H3, where its approach angle sterically blocks ACE2 from engaging the RBS. Virus neutralization by COVA1-16 is driven by IgG avidity.

## Introduction

Human infection with severe acute respiratory syndrome coronavirus 2 (SARS-CoV-2) (Zhou et al., 2020b) rapidly escalated to an ongoing global pandemic of coronavirus disease 2019 (COVID-19) (Kissler et al., 2020). Given the current lack of protective vaccines and antiviral agents, virus clearance and recovery from SARS-CoV-2 have to rely mainly on generation of a neutralizing antibody response. To date, most neutralizing antibodies from convalescent individuals target the receptor-binding domain (RBD) on the trimeric spike (S) glycoprotein (Brouwer et al., 2020; Cao et al., 2020; Robbiani et al., 2020; Rogers et al., 2020; Zost et al., 2020), whose natural function is to mediate viral entry by first attaching to the human receptor angiotensin-converting enzyme 2 (ACE2) and then fusing its viral membrane with the host cell (Lan et al., 2020; Letko et al., 2020; Shang et al., 2020; Yan et al., 2020; Zhou et al., 2020b).

SARS-CoV-2 is phylogenetically closely related to SARS-CoV (Zhou et al., 2020b), which caused the 2002–2003 human epidemic. However, SARS-CoV-2 and SARS-CoV only share 73% amino acid sequence identity in their RBD compared with 90% in their S2 fusion domain. Nevertheless, a highly conserved epitope on the SARS-CoV-2 RBD has been identified previously from studies of a SARS-CoV-neutralizing antibody, CR3022 (Tian et al., 2020; Yuan et al., 2020b), which was originally isolated almost 15 years ago (ter Meulen et al., 2006). Many human monoclonal antibodies have now been shown to target the SARS-CoV-2 S protein (Andreano et al., 2020; Barnes et al., 2020; Brouwer et al., 2020; Cao et al., 2020; Chi et al., 2020; Ju et al., 2020; Li et al., 2020; Liu et al., 2020; Pinto et al., 2020; Robbiani et al., 2020; Rogers et al., 2020; Seydoux et al., 2020; Shi et al., 2020; Wec et al., 2020; Wu et al., 2020c; Yuan et al., 2020b; Zost et al., 2020), but cross-neutralizing antibodies are relatively uncommon in individuals with COVID-19 (Brouwer et al., 2020; Ju et al., 2020; Lv et al., 2020; Rogers et al., 2020). To date, the only structurally characterized cross-neutralizing human antibodies are S309 (Pinto et al., 2020) and ADI-56046 (Wec et al., 2020) from SARS-CoV survivors as well as EY6A from an individual with COVID-19 (Zhou et al., 2020a). Such structural and molecular characterization of cross-neutralizing antibodies is extremely valuable to understand how to confer broader protection against human SARS-like viruses that include the extensive reservoir of zoonotic sarbecoviruses in bats, pangolins, etc.

Here we determined the crystal structure of a cross-neutralizing human antibody, COVA1-16, in complex with the SARS-CoV-2 RBD. COVA1-16 utilizes a long complementarity-determining region (CDR) H3 to target a highly conserved epitope and can cross-neutralize SARS-CoV. Although its epitope does not overlap with the ACE2 receptor binding site, COVA1-16 is able to compete with ACE2 for binding to the RBD. Our binding experiments and neutralization assays revealed that bivalent immunoglobulin G (IgG) binding is important for the neutralization activity of COVA1-16. We also performed a structural analysis to understand the functional constraints that underlie sequence conservation of the COVA1-16 epitope. This study provides insights into vaccine and therapy development for SARS-CoV-2 as well as other SARS-like viruses.

## Results

### COVA1-16 Binds to a Conserved Epitope on the SARS-CoV-2 RBD that Overlaps with the CR3022 Epitope

**Figure 1:**
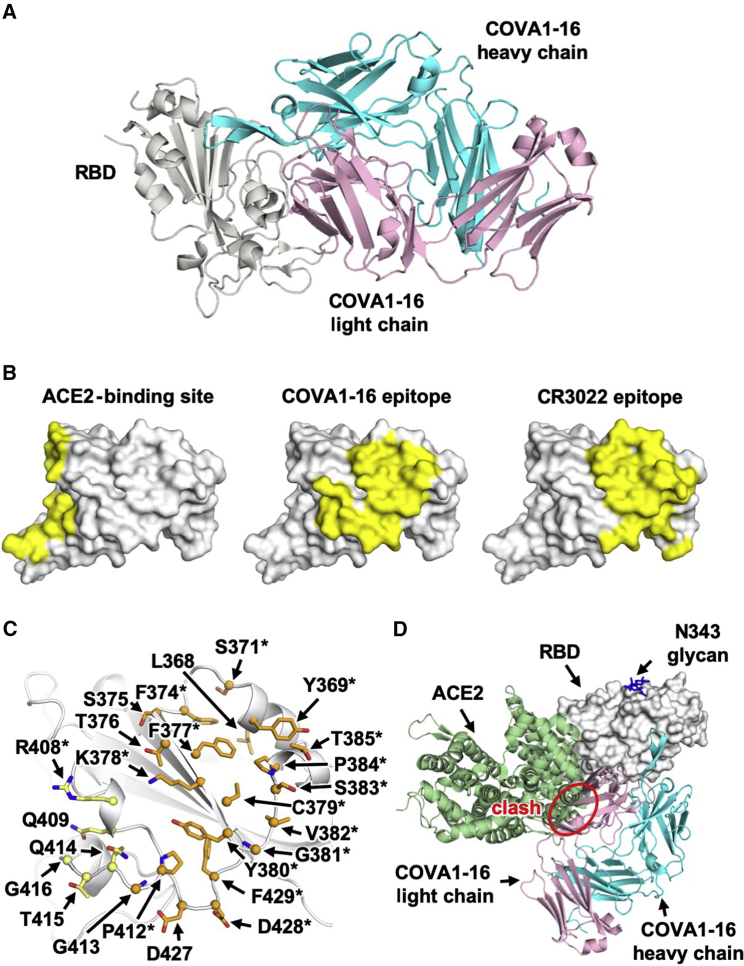
Comparison of the COVA1-16 Binding Mode with CR3022 and ACE2 (A) Crystal structure of the COVA1-16–RBD complex with the RBD in gray and the COVA1-16 Fab in cyan (heavy chain) and pink (light chain). (B) The ACE2-binding site (PDB: 6M0J; left; Lan et al., 2020), the COVA1-16 epitope (this study, center), and the CR3022 epitope (PDB: 6W41; right; Yuan et al., 2020b) are highlighted in yellow. (C) RBD residues in the COVA1-16 epitope. Epitope residues contacting the heavy chain are shown in orange and the light chain in yellow. Representative epitope residues are labeled. Residues that are also part of the CR3022 epitope are indicated by asterisks. (D) The ACE2-RBD complex structure is aligned in the same orientation as the COVA1-16-RBD complex. The COVA1-16 light chain (pink) would clash with ACE2 (green) if they were to approach their respective RBD binding sites at the same time (indicated by a red circle). See also Figure S1 and Tables S1 and S2.

The antibody COVA1-16 was recently isolated from an individual recovering from COVID-19 and cross-neutralizes SARS-CoV-2 (half-maximal inhibitory concentration [IC_50_], 0.13 μg/mL) and SARS-CoV (IC_50_, 2.5 μg/mL) pseudovirus (Brouwer et al., 2020). The heavy and light chains of COVA1-16 are encoded by immunoglobulin (IG) genes IGHV1-46, IGHD3-22, IGHJ1, IGKV1-33, and IGKJ4, with a relatively long CDR H3 of 20 amino acids (Figure S1). IGHV of COVA1-16 is only 1% somatically mutated in its nucleotide sequence (one amino acid change) from the germline gene, whereas its IGKV is 1.4% somatically mutated (three amino acid changes). Here we determined the crystal structure of COVA1-16 in complex with the SARS-CoV-2 RBD at 2.89-Å resolution to identify its binding site (epitope) and mechanism of cross-neutralization (Figure 1A; Table S1). The epitope of COVA1-16 overlaps extensively with that of CR3022 but also extends toward the periphery of the ACE2-binding site (Figure 1B; Yuan et al., 2020b). Seventeen of 25 residues in the COVA1-16 epitope overlap with the highly conserved CR3022 binding site (17 of 28 residues) (Figure 1C). Consistent with structural identification of its epitope, COVA1-16 can compete with CR3022 for RBD binding (Figure S2). COVA1-16 appears to have some resemblance to the SARS-CoV cross-neutralizing antibody ADI-56046, whose epitope appears to span the CR3022 epitope and ACE2-binding site, as indicated by negative-stain electron microscopy (nsEM) (Wec et al., 2020). COVA1-16 also competes with ACE2 for RBD binding (Figure S2; Brouwer et al., 2020), although its epitope does not overlap the ACE2-binding site (Figure 1B). Therefore, COVA1-16 inhibits ACE2 binding because of steric hindrance with its light chain rather than by direct interaction with the receptor binding site (Figure 1D).

### The Neutralization Activity of COVA1-16 Is Promoted by IgG Bivalent Binding

**Figure 2:**
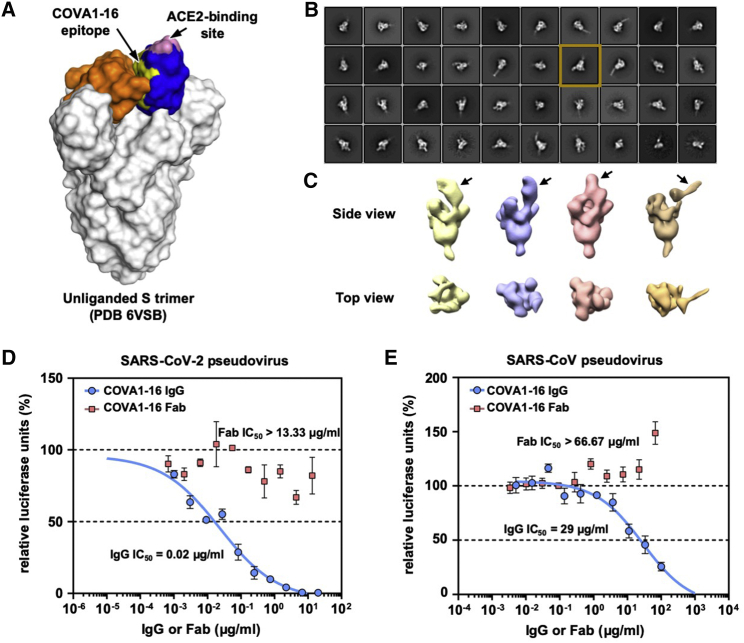
nsEM Analysis and IgG Avidity Effect of COVA1-16 (A) The COVA1-16 epitope on the unliganded SARS-CoV-2 S trimer with one RBD in the up conformation (blue) and two in the down conformation (orange) (PDB: 6VSB; Wrapp et al., 2020b). The COVA1-16 epitope is shown in yellow and the ACE2-binding site in pink. (B) Representative 2D class averages derived from thousands of single-particle images from nsEM analysis of a SARS-CoV-2 S trimer complexed with the COVA1-16 Fab for a single experiment. The 2D class corresponding to the most outward conformation of the COVA-16 Fab in complex with the S trimer is highlighted in a mustard box. (C) Various conformations of the COVA1-16 Fab in complex with the S trimer is revealed by 3D reconstruction. The location of the COVA1-16 Fab is indicated by an arrow. (D and E) Neutralization activities of COVA1-16 IgG (blue) and the Fab (red) against (D) SARS-CoV-2 and (E) SARS-CoV are measured in a luciferase-based pseudovirus assay. The half-maximal inhibitory concentration (IC_50_) values for IgG and the Fab are indicated in parentheses. Of note, neutralization of IgG (IC_50_ = 0.02 μg/mL) against the SARS-CoV-2 pseudovirus infecting 293T-ACE2 cells is comparable with that measured in Huh7 cells (IC_50_ = 0.13 μg/mL), as reported previously (Brouwer et al., 2020). Error bars indicate SEM of three technical replicates. See also Figures S2 and S3.

The RBD can adopt up and down conformations on the S trimer (Ke et al., 2020; Wrapp et al., 2020b). Although the ACE2 receptor only binds the RBD in the up conformation (Yan et al., 2020), the previously characterized cross-neutralizing antibody S309 from an individual recovering from SARS-CoV and COVA2-15 from an individual with SARS-CoV-2 (Brouwer et al., 2020) can bind the RBD in the up and down conformations (Pinto et al., 2020; Wrapp et al., 2020b). However, unlike S309, the COVA1-16 epitope is completely buried when the RBD is in the down conformation (Figure 2A), akin to the CR3022 epitope (Yuan et al., 2020b). Even in the up conformation of the RBD on an unliganded SARS-CoV-2 S trimer (Wrapp et al., 2020b), the epitope of COVA1-16 would not be fully exposed (Figure 2A). We thus performed nsEM analysis of COVA1-16 in complex with the SARS-CoV-2 S trimer (Figure 2B). Three-dimensional (3D) reconstructions revealed that COVA1-16 can bind to a range of RBD orientations on the S protein when in the up position, indicating its rotational flexibility (Figure 2C). COVA1-16 could bind the S trimer from the top (i.e., perpendicular to the trimer apex; Figure 2C, yellow, blue, and pink) or from the side (i.e., more tilted; Figure 2C, brown). Model fitting of the COVA1-16-RBD crystal structure into the nsEM map indicated that the RBD on the S trimer is more open around the apex when COVA1-16 binds compared with unliganded trimers (Figures S2B and S2C). Bivalent binding of the COVA1-16 IgG between adjacent S trimers also appeared to be plausible (Figure S2D). A recent cryoelectron tomography (cryo-ET) analysis demonstrated that the average distance between prefusion S on the viral surface is around 150 Å (Yao et al., 2020), which is comparable with the distance between the tip of the two antibody fragments (Fabs) on an IgG (typically around 100–150 Å, although longer distances have been observed) (Klein and Bjorkman, 2010). Indeed, COVA1-16 IgG bound much more tightly than the Fab to the SARS-CoV-2 RBD, with dissociation constant (K_D_) values of 0.2 nM and 46 nM, respectively (Figure S3A), reflecting bivalent binding in the assay format. Similarly, COVA1-16 IgG bound more strongly than the Fab to the SARS-CoV RBD (K_D_ of 125 nM versus 405 nM) (Figure S3B). Moreover, the apparent affinity of COVA1-16 IgG decreased to approximately the Fab value when the amount of SARS-CoV-2 RBD loaded on the biosensor was decreased, substantiating the notion that COVA1-16 can bind bivalently in this assay via interspike cross-linking (Figure S3C).

Bivalent IgG binding was also important for the neutralization activity of COVA1-16 (Figures 2D and 2E). COVA1-16 IgG neutralized SARS-CoV-2 pseudovirus with a half-maximal inhibitory concentration (IC_50_) of 0.02 μg/mL, which is similar to that measured previously measured for SARS-CoV-2 pseudovirus (IC_50_ of 0.13 μg/mL) (Brouwer et al., 2020). In contrast, the COVA1-16 Fab did not neutralize SARS-CoV-2 pseudovirus even up to 13 μg/mL. A similar effect was also observed for SARS-CoV pseudovirus, which was neutralized by COVA1-16 IgG at an IC_50_ of 29 μg/mL but not by the COVA1-16 Fab even up to 67 μg/mL (Figure 2E). Of note, COVA1-16 is less potent against authentic SARS-CoV-2 (IC_50_ = 0.75 μg/mL) (Brouwer et al., 2020). Whether such a difference is due to variation in S protein density on the viral surface versus pseudovirus or due to other factors deserves future investigation. It will also be informative to compare the number, density, and conformational states of the S proteins on SARS-CoV-2 and SARS-CoV virions. Our findings support the importance of bivalent binding for SARS-CoV-2-neutralizing antibodies and especially for cross-neutralization of SARS-CoV. Such a contribution of bivalent IgG (avidity) to SARS-CoV-2 neutralization has also been suggested in a recent study that compared binding of polyclonal IgGs and Fabs (Barnes et al., 2020). Furthermore, a single-domain camelid antibody, VHH-72, improved its neutralization activity to SARS-CoV-2 when expressed as a bivalent Fc fusion (Wrapp et al., 2020a). These observations are similar to some broadly neutralizing influenza antibodies to the hemagglutinin (HA) receptor binding site, where bivalent binding can increase avidity and neutralization breadth (Ekiert et al., 2012; Lee et al., 2012). Nevertheless, we have shown recently that the CR3022 Fab and IgG have similar neutralization potency as a SARS-CoV-2 variant with enhanced binding affinity for CR3022 (Wu et al., 2020a), suggesting that an avidity effect is not universally observed for all RBD-targeting antibodies, especially to this particular epitope, which is targeted by CR3022 and COVA1-16.

### Binding of COVA1-16 to the RBD Is Dominated by CDR H3

**Figure 3:**
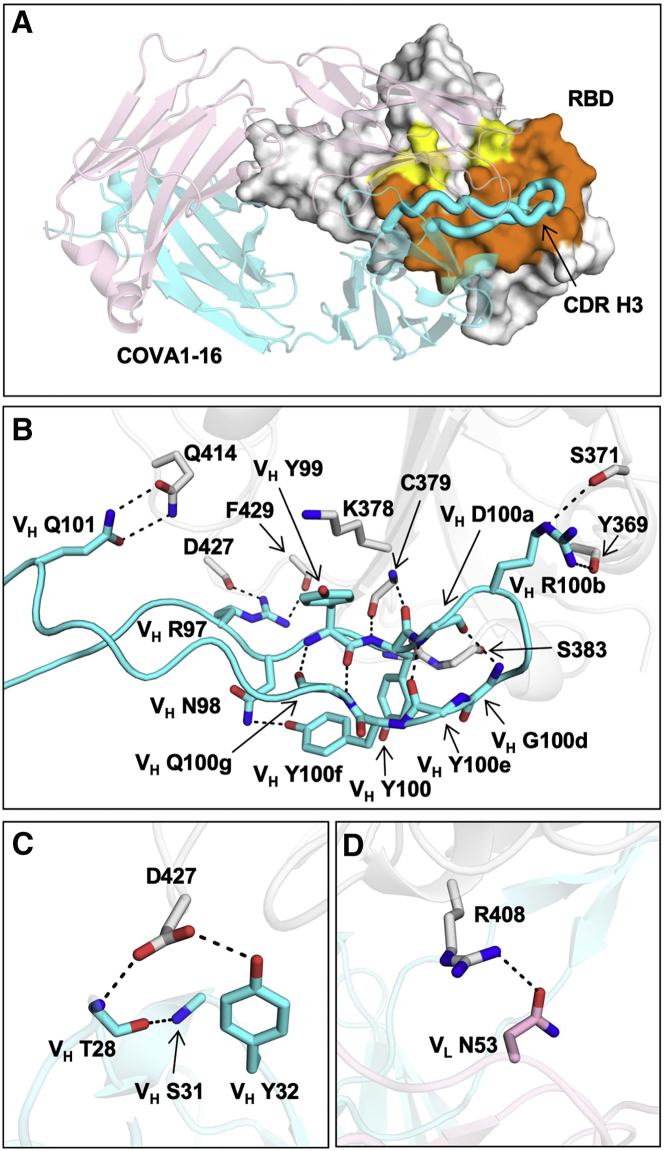
Interaction between the SARS-CoV-2 RBD and COVA1-16 (A) The epitope of COVA1-16 is highlighted in yellow and orange. Epitope residues that are in contact with CDR H3 are shown in orange and in yellow otherwise. COVA1-16 (heavy chain in cyan and light chain in pink) is in cartoon representation, with CDR H3 depicted as a thick tube. The RBD (white) is in a surface representation. The BSAs on COVA1-16 and RBD are 827 Å^2^ and 780 Å^2^, respectively. (B–D) Interactions of the SARS-CoV-2 RBD (white) with (B) CDR H3 (cyan), (C) CDR H1 (cyan), and (D) CDR L2 (pink) of COVA1-16. H-bonds are represented by dashed lines. In (C), a 3_10_ turn is observed in CDR H1 for residues V_H_ T28–V_H_ S31. See also Figures S2 and S3.

Next we examined the molecular details of the interactions between COVA1-16 and SARS-CoV-2. COVA1-16 binding to the RBD is dominated by the heavy chain, which accounts for 81% of its total buried surface area (BSA; 673 Å^2^ of a total of 827 Å^2^). Most of the interactions are mediated by CDR H3 (Figure 3A), which contributes 72% (594 Å^2^) of the total BSA. CDR H3 forms a beta-hairpin with a type I beta-turn at its tip and is largely encoded by IGHD3-22 (from N98 to heavy chain variable domain (V_H_) Y100f; Figure S1C; Figure 3B). The beta-hairpin conformation is stabilized by four main chain-main chain hydrogen bonds (H-bonds) and a side chain-side chain H-bond between V_H_ N98 and V_H_ Y100f at either end of the IGHD3-22-encoded region (Figure 3B). Four H-bonds between the tip of CDR H3 and the RBD are formed from two main chain-main chain interactions with RBD C379 and two with V_H_ R100b (Table S2). The positively charged guanidinium of V_H_ R100b also interacts with the partial negative dipole at the C terminus of a short α helix in the RBD (residues Y365–Y369). V_H_ R100b is a somatically mutated residue (codon = AGG in the IGHD3-22-encoded region, where the germline residue is a Ser [codon = AGT]; Figure S1C). The short Ser side chain would likely not contact the RBD or provide electrostatic complementarity. A somatic revertant V_H_ R100bS actually improved the binding affinity of COVA1-16 to the RBD, mostly because of an increased on rate (Figure S3D). Nevertheless, COVA1-16 has a much slower off rate than its V_H_ R100bS mutant, which may have led to its selection. The CDR H3 tip also interacts with the RBD through hydrophobic interactions between V_H_ Y99 and the aliphatic portion of RBD K378 as well as a π-π interaction between V_H_ Y100 and the RBD V382-S383 peptide backbone (Figure 3B). CDR H3 forms an additional four H-bonds with the RBD, involving the side chains of V_H_ R97 and Q101 (Figure 3B). We further determined the unliganded structure of COVA1-16 Fab to 2.53-Å resolution and found that the CDR H3 distal region was not resolved because of lack of electron density, indicating its inherent flexibility (Figures S2E and S2F). CDR H1 and CDR L2 of COVA1-16 also interact with the RBD, but much less so compared with CDR H3. The V_H_ T28 main chain and V_H_ Y32 side chain in CDR H1 H-bond with D427 (Figure 3C; Table S2), whereas V_L_ N53 in CDR L2 H-bonds with RBD R408 (Figure 3D; Table S2).

Although SARS-CoV-2 and SARS-CoV differ by only two amino acid residues (A372T and P384A) in the COVA1-16 epitope (Figure S4), they do not appear to account for the affinity differences in COVA1-16 binding to the RBD (Figure S3E). As a result, the binding affinity of COVA1-16 to the RBD may be influenced by residues outside of the epitope as well as the dynamics of the RBD fluctuations between up and down conformations.

### Sequence Conservation of the COVA1-16 Epitope Appears to Arise from Functional Constraints in the S Protein

**Figure 4:**
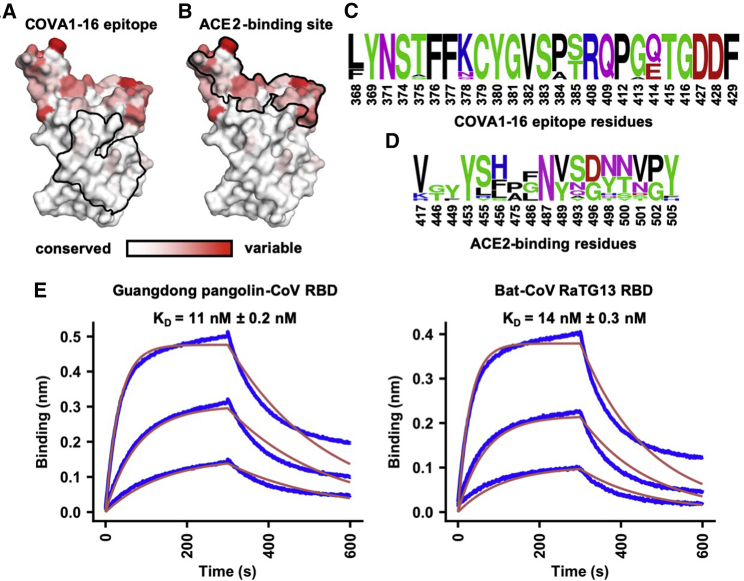
Sequence Conservation of the COVA1-16 Epitope and ACE2-Binding Site (A and B) Sequence conservation of the RBD among 17 SARS-like CoVs (Figure S4) is highlighted on the RBD structure, with (A) COVA1-16 epitope and (B) ACE2-binding site indicated by the black outline. The backside of this view is shown in Figure S5A. (C and D) Sequence conservation of the (C) COVA1-16 epitope and (D) ACE2-binding site is shown as a sequence logo. (E) The binding kinetics of COVA1-16 IgG to RBDs from Guangdong pangolin CoV and bat CoV RaTG13 were measured by biolayer interferometry (BLI) with IgG on the biosensor and RBD in solution. The y axis represents the response. Dissociation constant (K_D_) values were obtained using a 1:1 binding model and are represented by the red lines. Representative results of two replicates for each experiment are shown. See also Figures S4 and S5.

**Figure 5:**
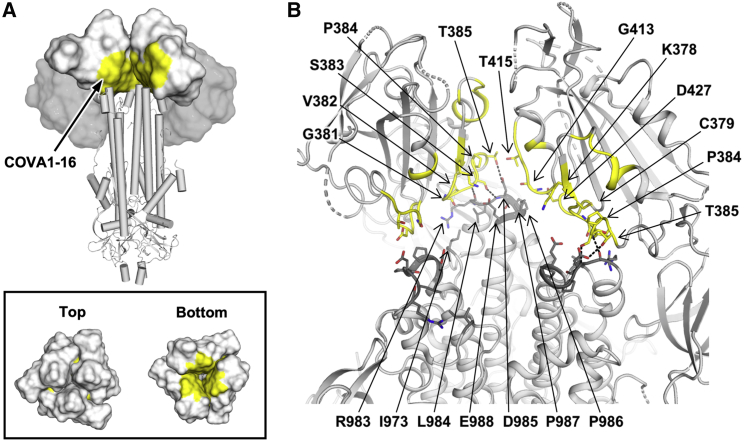
Structural and Functional Constraints of the COVA1-16 Epitope (A) Location of the COVA1-16 epitope (yellow) on the SARS-CoV-2 S trimer when all three RBDs are in the down conformation (PDB: 6VXX; Walls et al., 2020). RBDs are represented as a white surface, N-terminal domains (NTDs) as a gray surface, and the S2 domain in a cartoon representation. Top panel: for visualization of the COVA1-16 epitope, the RBD and NTD from one of the three protomers was removed. Bottom panel: top and bottom views of the COVA1-16 epitopes on the three RBDs in the down conformation. (B) The COVA1-16 epitope is shown in yellow on a ribbon representation of a SARS-CoV-2 S trimer (PDB: 6VXX; Walls et al., 2020). Epitope residues in the RBD involved in interaction with the S2 domain are shown as yellow sticks and S2 domain-interacting residues as dark gray sticks. Dashed lines indicate H-bonds. Interface residues were calculated using PISA software (Krissinel and Henrick, 2007). The S1 segment from the third protomer is omitted to clarify the view of the interfaces the COVA1-16 epitope makes with the S2 domain. See also Figure S5.

Compared with the ACE2-binding site, the COVA1-16 epitope is much more highly conserved among SARS-CoV-2, SARS-CoV, and other SARS-related coronaviruses (SARSr-CoVs) (Figures 4A–4D; Figures S4 and S5A; Brouwer et al., 2020). Consistent with the sequence conservation of the epitope, COVA1-16 could bind to RBDs from Guangdong pangolin CoV and bat CoV RaTG13 (Figure 4E). To investigate possible structural and functional reasons for this sequence conservation, we analyzed the epitope location in the context of the SARS-CoV-2 trimeric S protein with all RBDs in the down conformation (Walls et al., 2020; Figure 5A; Figure S4). The COVA1-16 epitope is completely buried at the center of the trimer in the interface between the S1 and S2 domains and is largely hydrophilic (Figure S5B). The polar side chains of K378, Q414, R408, and D427, which are involved in binding to COVA1-16, are all very close to the interface with adjacent protomers in the S trimer. The R408 side chain, which is positioned by Q414 via an H-bond, points toward a region in the adjacent protomer 2 with a positive electrostatic potential. Similarly, D427 is juxtaposed to a region in protomer 2 with a negative electrostatic potential. These repulsive charges would help favor the metastability required for transient opening and closing of the RBD in up and down conformations prior to ACE2 receptor binding. In contrast, the K378 side chain points toward a region in protomer 3 with negative electrostatic potential, favoring the down RBD conformation. Furthermore, in the down conformation, part of the COVA1-16 epitope interacts with the long helices formed by the heptad repeat motifs of the S2 fusion domain (Figures 5A and 5B). Notably, S383 and T385 in the COVA1-16 epitope make three H-bonds with the tops of the helices and their connecting regions (Figure 5B). This mixture of attractive and repulsive forces would seem to be important for control of the dynamics of the RBD and, hence, for the biological function of the metastable pre-fusion S protein in receptor binding and fusion. The complementarity of fit of the epitope interface with the other RBDs and the S2 domain in the S trimer further explains the epitope conservation (Figures S5C–S5G). Therefore, the high sequence conservation of the COVA1-16 epitope appears to be related to the functional requirement for this component of the RBD surface to be deeply buried within the S trimer in the down conformation. The COVA1-16 epitope and its interaction with the RBD on the S compared with other antibody epitopes is illustrated in Figure S5H. Only a small part of the RBD surface (“silent face”) has not yet been observed to bind antibodies to SARS-CoV-2.

## Discussion

From the SARS-CoV-2 RBD-antibody complex structures to date, a substantial portion of the RBD surface can be targeted by antibodies (Yuan et al., 2020c). One surface not yet observed to be targeted is partially covered by N-glycans at residues N165 on the N-terminal domain (NTD) and N343 on the RBD (Watanabe et al., 2020), which may hinder B cell receptor access and create a silent face, although the N343 glycan is incorporated in the S309 epitope (Pinto et al., 2020). While antibodies that target the ACE2-binding site, such as BD23 (Cao et al., 2020), CB6 (Shi et al., 2020), B38 (Wu et al., 2020c), P2B-2F6 (Ju et al., 2020), CC12.1 (Yuan et al., 2020a), CC12.3 (Yuan et al., 2020a), COVA2-04 (Wu et al., 2020b), and COVA2-39 (Wu et al., 2020b), do not show cross-neutralization activity to SARS-CoV, conserved epitopes that seem to be more able to support cross-neutralization can be found elsewhere (Pinto et al., 2020; Yuan et al., 2020b; Zhou et al., 2020a). So far, these rare cross-neutralizing antibodies, including COVA1-16, often seem to bind to epitopes that are not readily accessible in the pre-fusion native structure when the RBD is in the down conformation (Wec et al., 2020; Zhou et al., 2020a). This finding is similar to a recent discovery in influenza virus, where a class of cross-protective antibodies target a conserved epitope in the trimeric interface of the HA (Bajic et al., 2019; Bangaru et al., 2019; Watanabe et al., 2019). Because of the inaccessibility of the COVA1-16 epitope on the S protein, it is possible that an RBD-based rather than an S-based immunogen can elicit larger numbers of COVA1-16-like antibodies.

A main feature of COVA1-16 is its CDR H3-dominant binding mode. In fact, CDR H3-dominant antibodies have been seen in the human immune response to other viral pathogens. Some pertinent examples are the antibodies PG9 and PG16, whose CDR H3s interact extensively along their length with the apex of the HIV-1 Envelope protein (McLellan et al., 2011; Pan et al., 2020). Another example is C05, which is essentially a single loop binder that inserts its very long CDR H3 (24 residues) into the RBD of influenza HA (Ekiert et al., 2012), providing a template for design of a high-avidity protein inhibitor of influenza virus, where the H3 loop is fused to a scaffold protein (Strauch et al., 2017). The long CDR H3 of COVA1-16 may similarly facilitate therapeutic designs that could also include peptide-based antiviral agents, as exemplified by a potent cyclic peptide fusion inhibitor of influenza HA (Corti et al., 2011; Kadam et al., 2017).

As SARS-CoV-2 continues to circulate in the human population, and other zoonotic coronaviruses constitute future pandemic threats (Menachery et al., 2015), it is certainly worth considering development of more universal coronavirus vaccines and therapeutic agents that can cross-neutralize antigenically drifted SARS-CoV-2 viruses as well as zoonotic SARS-like coronaviruses. This process will highly benefit from continued characterization of cross-neutralizing antibodies, as demonstrated for influenza virus (Wu and Wilson, 2018) and HIV (Ward and Wilson, 2020).

## STAR★Methods

### Key Resources Table

**Table:** 

REAGENT or RESOURCE	SOURCE	IDENTIFIER
ExpiCHO Expression System Kit	Thermo Fisher Scientific	A29133
Expi293 Expression System Kit	Thermo Fisher Scientific	A14635
HyClone insect cell culture medium	GE Healthcare	SH30280.03
FreeStyle 293 expression medium	GIBCO	12338002
Opti-MEM I reduced serum media	GIBCO	51985091
Phosphate-buffered saline (PBS)	Thermo Fisher Scientific	14040133
Ni-NTA Superflow	QIAGEN	30450
DH10Bac competent cells	Thermo Fisher Scientific	10361012
CaptureSelect CH1-XL Affinity Matrix	Thermo Fisher Scientific	2943452010
Protein A column	Thermo Fisher Scientific	17040301

**Chemicals and Recombinant Proteins**		

DpnI	New England Biolabs	R0176L
Trypsin	New England Biolabs	P8101S
Fugene 6 Transfection Regent	Promega	E2691
BirA Biotin-Protein Ligase Reaction Kit	Avidity	BIRA-500
Sodium chloride (NaCl)	Sigma-Aldrich	S9888
Tris Base	Sigma-Aldrich	11814273001
Concentrated hydrochloric acid (HCl)	Sigma-Aldrich	H1758
Sodium azide (NaN_3_)	Sigma-Aldrich	S2002
Bovine Serum Albumin (BSA)	Sigma-Aldrich	A9418
Tween 20	Fisher Scientific	BP337-500
PEImax	Polysciences	24765-1
Chemicals for protein crystallization	Hampton Research	N/A

**Critical Commercial Assays**		

In-Fusion HD Cloning Kit	Takara	639647
KOD Hot Start DNA Polymerase	EMD Millipore	71086-3
PCR Clean-Up and Gel Extraction Kit	Clontech Laboratories	740609.250
QIAprep Spin Miniprep Kit	QIAGEN	27106
NucleoBond Xtra Maxi	Clontech Laboratories	740414.100

**Deposited Data**		

X-ray coordinates and structure factors of COVA1-16 Fab	This study	PDB: 7JMX
X-ray coordinates and structure factors of COVA1-16 Fab in complex with SARS-CoV-2 RBD	This study	PDB: 7JMW

**Cell Lines**		

ExpiCHO cells	Thermo Fisher Scientific	A29127
Expi293F cells	Thermo Fisher Scientific	A14527
HEK293F cells	Invitrogen	R79007
Sf9 cells	ATCC	CRL-1711
High Five cells	Thermo Fisher Scientific	B85502

**Recombinant DNA**		

phCMV3-COVA1-16 IgG heavy chain	Brouwer et al., 2020	N/A
phCMV3-COVA1-16 Fab heavy chain	Brouwer et al., 2020	N/A
phCMV3-COVA1-16 light chain	Brouwer et al., 2020	N/A
pPPI4-SARS-CoV RBD	Brouwer et al., 2020	N/A
pPPI4-SARS-CoV-2 RBD	Brouwer et al., 2020	N/A
pPPI4-SARS-CoV	Brouwer et al., 2020	N/A
pPPI4-SARS-CoV-2	Brouwer et al., 2020	N/A
pFastBac-SARS-CoV-RBD	Yuan et al., 2020b	N/A
pFastBac-SARS-CoV-2-RBD	Yuan et al., 2020b	N/A
phCMV3-ACE2	This study	N/A

**Software and Algorithms**		

HKL2000	Otwinowski and Minor, 1997	N/A
Phaser	McCoy et al., 2007	N/A
Coot	Emsley et al., 2010	N/A
Phenix	Adams et al., 2010	N/A
MolProbity	Chen et al., 2010	N/A
WebLogo	Crooks et al., 2004	N/A
PyMOL	Schrödinger, LLC	RRID:SCR_000305
Appion	Lander et al., 2009	N/A
DoG Picker	Voss et al., 2009	N/A
Relion	Zivanov et al., 2018	N/A
UCSF Chimera	Pettersen et al., 2004	N/A
Octet analysis software 9.0	Fortebio	https://www.moleculardevices.com/

**Other**		

Fab-CH1 2nd generation (FAB2G) biosensors	ForteBio	Cat# 18-5019
Ni-NTA biosensors	ForteBio	Cat# 18-5102
Streptavidin (SA) biosensors	ForteBio	Cat# 18-5020
Negative stain EM grids, 400 mesh	Electron Microscopy Sciences	EMS400-CU

### Resource Availability

#### Lead Contact

Information and requests for resources and reagents should be directed to and will be fulfilled by the Lead Contact, Ian A. Wilson (wilson@scripps.edu).

#### Materials Availability

All reagents generated in this study are available from the Lead Contact (I.A.W.) with a completed Materials Transfer Agreement.

#### Data and Code Availability

X-ray coordinates and structure factors have been deposited in the RCSB Protein Data Bank under accession codes PDB: 7JMW and 7JMX. COVA1-16 IGVH and IGVK sequences are available in GenBank: MT599835 and MT599919. The EM maps have been deposited at the Electron Microscopy Data Bank (EMDB) with accession codes EMDB: EMD-22742, EMD-22743, EMD-22744, and EMD-22745.

### Experimental Model and Subject Details

#### Expression and Purification of SARS-CoV, SARS-CoV-2, and SARSr-CoV RBDs

The receptor-binding domain (RBD) (residues 319-541) of the SARS-CoV-2 spike (S) protein (GenBank: QHD43416.1), the RBD (residues 306-527) of the SARS-CoV S protein (GenBank: ABF65836.1), the RBD (residues 315-537) of pangolin-CoV (GenBank: QLR06866.1), and the RBD (residues 319-541) of Bat-CoV RaTG13 (GenBank: QHR63300.2) were cloned into a customized pFastBac vector (Ekiert et al., 2011), and fused with an N-terminal gp67 signal peptide and C-terminal His_6_ tag (Yuan et al., 2020b). For each RBD, we further cloned a construct with an AviTag inserted in front of the His_6_ tag. To express the RBD, a recombinant bacmid DNA was generated using the Bac-to-Bac system (Life Technologies). Baculovirus was generated by transfecting purified bacmid DNA into Sf9 cells using FuGENE HD (Promega), and subsequently used to infect suspension cultures of High Five cells (Life Technologies) at an MOI of 5 to 10. Infected High Five cells were incubated at 28 °C with shaking at 110 rpm for 72 h for protein expression. The supernatant was then concentrated using a 10 kDa MW cutoff Centramate cassette (Pall Corporation). The RBD protein was purified by Ni-NTA, followed by size exclusion chromatography, and buffer exchanged into 20 mM Tris-HCl pH 7.4 and 150 mM NaCl. For binding experiments, RBD with AviTag was biotinylated as described previously (Ekiert et al., 2012) and purified by size exclusion chromatography on a Hiload 16/90 Superdex 200 column (GE Healthcare) in 20 mM Tris-HCl pH 7.4 and 150 mM NaCl.

#### Expression and Purification of COVA1-16 Fab

Expression plasmids encoding the heavy and light chains of the COVA1-16 Fab were transiently co-transfected into ExpiCHO cells at a ratio of 2:1 (HC:LC) using ExpiFectamine CHO Reagent (Thermo Fisher Scientific) according to the manufacturer’s instructions. The supernatant was collected at 10 days post-transfection. The Fabs were purified with a CaptureSelect CH1-XL Affinity Matrix (Thermo Fisher Scientific) followed by size exclusion chromatography.

#### Expression and Purification of ACE2

The N-terminal peptidase domain of human ACE2 (residues 19 to 615, GenBank: BAB40370.1) was cloned into phCMV3 vector and fused with a C-terminal Fc tag. The plasmids were transiently transfected into Expi293F cells using ExpiFectamine 293 Reagent (Thermo Fisher Scientific) according to the manufacturer’s instructions. The supernatant was collected at 7 days post-transfection. Fc-tagged ACE2 protein was then purified with a Protein A column (GE Healthcare) followed by size exclusion chromatography.

#### Crystallization and X-ray Structure Determination

The COVA1-16 Fab complex with RBD was formed by mixing each of the protein components in an equimolar ratio and incubating overnight at 4°C. The COVA1-16 Fab–RBD complex and COVA1-16 Fab apo (unliganded) protein were adjusted to around 11 mg/mL and screened for crystallization using the 384 conditions of the JCSG Core Suite (QIAGEN) on our custom-designed robotic CrystalMation system (Rigaku) at Scripps Research. Crystallization trials were set-up by the vapor diffusion method in sitting drops containing 0.1 μL of protein and 0.1 μL of reservoir solution. Crystals used for X-ray data collection were harvested from drops containing 0.2 M sodium iodide and 20% (w/v) polyethylene glycol 3350 for the COVA1-16 Fab–RBD complex and from drops containing 0.08 M acetate pH 4.6, 20% (w/v) polyethylene glycol 4000, 0.16 M ammonium sulfate and 20% (v/v) glycerol for the COVA1-16 Fab. Crystals appeared on day 3, were harvested on day 7, pre-equilibrated in cryoprotectant containing 20% glycerol, and then flash cooled and stored in liquid nitrogen until data collection. Diffraction data were collected at cryogenic temperature (100 K) at Stanford Synchrotron Radiation Lightsource (SSRL) on the Scripps/Stanford beamline 12-1 with a beam wavelength of 0.97946 Å, and processed with HKL2000 (Otwinowski and Minor, 1997). Structures were solved by molecular replacement using PHASER (McCoy et al., 2007). The models for molecular replacement of RBD and COVA1-16 were from PDB: 6XC4 (Yuan et al., 2020a), 4IMK (Fenn et al., 2013) and 2Q20 (Baden et al., 2008). Iterative model building and refinement were carried out in COOT (Emsley et al., 2010) and PHENIX (Adams et al., 2010), respectively. Ramachandran statistics were calculated by MolProbity (Chen et al., 2010). Epitope and paratope residues, as well as their interactions, were identified by accessing PISA software server (https://www.ebi.ac.uk/pdbe/prot_int/pistart.html; Krissinel and Henrick, 2007).

#### Expression and Purification of Recombinant S Protein for Negative-Stain EM

The SARS-CoV-2 S construct used for negative-stain EM contains the mammalian-codon-optimized gene encoding residues 1-1208 of the S protein (GenBank: QHD43416.1), followed by a C-terminal T4 fibritin trimerization domain, an HRV3C cleavage site, 8x-His tag and a Twin-strep tags subcloned into the eukaryotic-expression vector pcDNA3.4. Three amino-acid mutations were introduced into the S1–S2 cleavage site (RRAR to GSAS) to prevent cleavage and two stabilizing proline mutations (K986P and V987P) to the HR1 domain. For additional S stabilization, residues T883 and V705 were mutated to cysteines to introduce a disulphide bond. The S plasmid was transfected into 293F cells and supernatant was harvested at 6 days post transfection. S protein was purified by running the supernatant through a streptactin column and then by size exclusion chromatography using a Superose 6 increase 10/300 column (GE Healthcare Biosciences). Protein fractions corresponding to the trimeric S protein were collected and concentrated.

#### nsEM Sample Preparation and Data Collection

SARS-CoV-2 S protein was complexed with 3x molar excess of Fab for 30 min prior to direct deposition onto carbon-coated 400-mesh copper grids. The grids were stained with 2% (w/v) uranyl-formate for 90 s immediately following sample application. Grids were either imaged at 200 keV or at 120 keV on a Tecnai T12 Spirit using a 4kx4k Eagle CCD. Micrographs were collected using Leginon (Suloway et al., 2005) and the images were transferred to Appion for processing. Particle stacks were generated in Appion (Lander et al., 2009) with particles picked using a difference-of-Gaussians picker (DoG-picker) (Voss et al., 2009). Particle stacks were then transferred to Relion (Zivanov et al., 2018) for 2D classification followed by 3D classification to sort well-behaved classes. Selected 3D classes were auto-refined on Relion and used to make figures with UCSF Chimera (Pettersen et al., 2004). A published prefusion spike model (PDB: 6Z97) (Huo et al., 2020) was used in our structural analysis.

#### Protein Expression and Purification for Antibody Binding Studies

All constructs were expressed transiently in HEK293F (Invitrogen, cat no. R79009) cells maintained in Freestyle medium (Life Technologies). For soluble RBD proteins, cells were transfected at a density of 0.8-1.2 million cells/mL by addition of a mix of PEImax (1 μg/μL) with expression plasmids (312.5 μg/L) in a 3:1 ratio in OptiMEM. Supernatants of the soluble RBD proteins were harvested six days post transfection, centrifuged for 30 min at 4000 rpm and filtered using 0.22 μm Steritop filters (Merck Millipore). Constructs with a His_6_-tag were purified by affinity purification using Ni-NTA agarose beads. Protein eluates were concentrated, and buffer exchanged to PBS using Vivaspin filters with a 10 kDa molecular weight cutoff (GE Healthcare). Protein concentrations were determined by Nanodrop using the proteins peptidic molecular weight and extinction coefficient as determined by the online ExPASy software (ProtParam). For the COVA1-16 IgG1 antibody, suspension HEK293F cells (Invitrogen, cat no. R79007) were cultured in FreeStyle medium (GIBCO) and co-transfected with the two IgG plasmids expressing the corresponding HC and LC in a 1:1 ratio at a density of 0.8-1.2 million cells/mL in a 1:3 ratio with 1 mg/L PEImax (Polysciences). The recombinant IgG antibodies were isolated from the cell supernatant after five days as described previously (20, 48). In short, the cell suspension was centrifuged 25 min at 4000 rpm, and the supernatant was filtered using 0.22 μm pore size SteriTop filters (Millipore). The filtered supernatant was run over a 10 mL protein A/G column (Pierce) followed by two column volumes of PBS wash. The antibodies were eluted with 0.1 M glycine pH 2.5, into the neutralization buffer of 1 M TRIS pH 8.7 in a 1:9 ratio. The purified antibodies were buffer exchanged to PBS using 100 kDa VivaSpin20 columns (Sartorius). The IgG concentration was determined on the NanoDrop 2000 and the antibodies were stored at 4°C until further analyses.

#### Measurement of Binding Affinities Using Biolayer Interferometry

To determine the binding affinity of COVA1-16 IgG and His-tagged Fabs, 20 μg/mL of His-tagged SARS-CoV or SARS-CoV-2 RBD protein in running buffer (PBS, 0.02% Tween-20, 0.1% BSA) was loaded on Ni-NTA biosensors (ForteBio) for 300 s. Streptavidin biosensors (ForteBio) were used if the RBD was biotinylated. Next, the biosensors were transferred to running buffer containing IgG or Fab to determine the association rate, after which the sensor was transferred to a well containing running buffer to allow dissociation. As negative control, an anti-HIV-1 His-tagged Fab was tested at the highest concentration used for COVA1-16 Fab (400 nM). After each cycle, the sensors were regenerated by alternating 20 mM glycine in PBS and running buffer three times, followed by reactivation in 20 mM NiCl_2_ for 120 s. All steps were performed at 1000 rpm shaking speed. K_D_s were determined using ForteBio Octet CFR software. The avidity effects of IgG were investigated by titrating the SARS-CoV-2 RBD concentration (5, 1, 0.2 and 0.04 μg/mL) followed by loading on Ni-NTA biosensors for 480 s with an additional loading step with His-tagged HIV-1 gp41 for 480 s to minimize background binding of His-tagged Fabs to the biosensor. All other steps were performed as described above.

#### Competition Studies of Antibodies with ACE2 Receptor

For competition assays, COVA1-16 IgG, CR3022 IgG, and human ACE2-Fc were all diluted to 250 nM. Ni-NTA biosensors were used. In brief, the assay has five steps: 1) baseline: 60 s with 1x kinetics buffer; 2) loading: 180 s with 20 μg/mL, His_6_-tagged SARS-CoV-2 RBD proteins; 3) baseline: 150 s with 1x kinetics buffer; 4) first association: 300 s with CR3022 IgG or human ACE2-Fc; and 5) second association: 300 s with human ACE2-Fc, CR3022 IgG, or COVA1-16 IgG.

#### Pseudovirus Neutralization Assay

Neutralization assays were performed using SARS-CoV and SARS-CoV-2 S-pseudotyped HIV-1 virus and HEK293T–ACE2 cells as described previously (Schmidt et al., 2020). In brief, pseudotyped virus was produced by co-transfecting expression plasmids of SARS-CoV S and SARS-CoV-2_Δ19_ S proteins (GenBank; AAP33697.1 and MT449663.1, respectively) with an HIV backbone expressing NanoLuc luciferase (pHIV-1_NL4-3_ ΔEnv-NanoLuc) in HEK293T cells (ATCC, CRL-11268). After 3 days, the cell culture supernatants containing SARS-CoV and SARS-CoV-2 S-pseudotyped HIV-1 viruses were stored at −80°C. HEK293T–ACE2 cells were seeded 10,000 cells/well in a 96-well plate one day prior to the start of the neutralization assay. To determine the neutralizing capacity of COVA1-16 IgG and His_6_-tagged Fab, 20 or 100 μg/mL COVA1-16 IgG and equal molar of COVA1-16 Fab were serially diluted in 3-fold steps and mixed with SARS-CoV or SARS-CoV-2 pseudotyped virus and incubated for 1 h at 37°C. The pseudotyped virus and COVA1-16 IgG or Fab mix were then added to the HEK293T–ACE2 cells and incubated at 37°C. After 48 h, cells were washed twice with PBS (Dulbecco’s Phosphate-Buffered Saline, eBiosciences) and lysis buffer was added. Luciferase activity of cell lysate was measured using the Nano-Glo Luciferase Assay System (Promega) and GloMax Discover System. The inhibitory concentration (IC_50_) was determined as the concentration of IgG or Fab that neutralized 50% of the pseudotyped virus using GraphPad Prism software (version 8.3.0).

#### Shape Complementarity Analysis

Shape complementarity values (Sc) were calculated as described by Lawrence and Colman (1993).

#### Sequence Conservation Analysis

RBD protein sequences from SARS-CoV and SARS-related coronavirus (SARSr-CoV) strains were retrieved from the following accession codes:

• GenBank ABF65836.1 (SARS-CoV)
• GenBank ALK02457.1 (Bat SARSr-CoV WIV16)
• GenBank AGZ48828.1 (Bat SARSr-CoV WIV1)
• GenBank ACU31032.1 (Bat SARSr-CoV Rs672)
• GenBank AIA62320.1 (Bat SARSr-CoV GX2013)
• GenBank AAZ67052.1 (Bat SARSr-CoV Rp3)
• GenBank AIA62300.1 (Bat SARSr-CoV SX2013)
• GenBank ABD75323.1 (Bat SARSr-CoV Rf1)
• GenBank AIA62310.1 (Bat SARSr-CoV HuB2013)
• GenBank AAY88866.1 (Bat SARSr-CoV HKU3-1)
• GenBank AID16716.1 (Bat SARSr-CoV Longquan-140)
• GenBank AVP78031.1 (Bat SARSr-CoV ZC45)
• GenBank AVP78042.1 (Bat SARSr-CoV ZXC21)
• GenBank QHR63300.2 (Bat CoV RaTG13)
• NCBI Reference Sequence YP_003858584.1 (Bat SARSr-CoV BM48-31)
• GISAID EPI_ISL_410721 (Pangolin BetaCoV Guandong2019)

Multiple sequence alignment of the RBD sequences was performed by MUSCLE version 3.8.31 (Edgar, 2004). Sequence logos were generated by WebLogo (Crooks et al., 2004). The conservation score of each RBD residue was calculated and mapped onto the SARS-CoV-2 RBD X-ray structure with ConSurf (Ashkenazy et al., 2016).

### Quantification and Statistical Analysis

Statistical analysis was not performed in this study.

## Supplemental Information

Document S1. Figures S1–S5 and Tables S1 and S2

Document S2. Article plus Supplemental Information
