# Supplementary material for: Cross-Neutralization of a SARS-CoV-2 Antibody to a Functionally Conserved Site Is Mediated by Avidity
Source: Immunity. 2020 Dec 15;53(6):1272–1280.e5. doi: 10.1016/j.immuni.2020.10.023 (PMC7687367; doi:10.1016/j.immuni.2020.10.023)
Supplement: Document S1. Figures S1–S5 and Tables S1 and S2 [file mmc1.pdf]

Immunity, Volume *53*

Supplemental Information

Cross-Neutralization of a SARS-CoV-2 Antibody to a Functionally Conserved Site Is Mediated by Avidity

Hejun Liu, Nicholas C. Wu, Meng Yuan, Sandhya Bangaru, Jonathan L. Torres, Tom G. Caniels, Jelle van Schooten, Xueyong Zhu, Chang-Chun D. Lee, Philip J.M. Brouwer, Marit J. van Gils, Rogier W. Sanders, Andrew B. Ward, and Ian A. Wilson

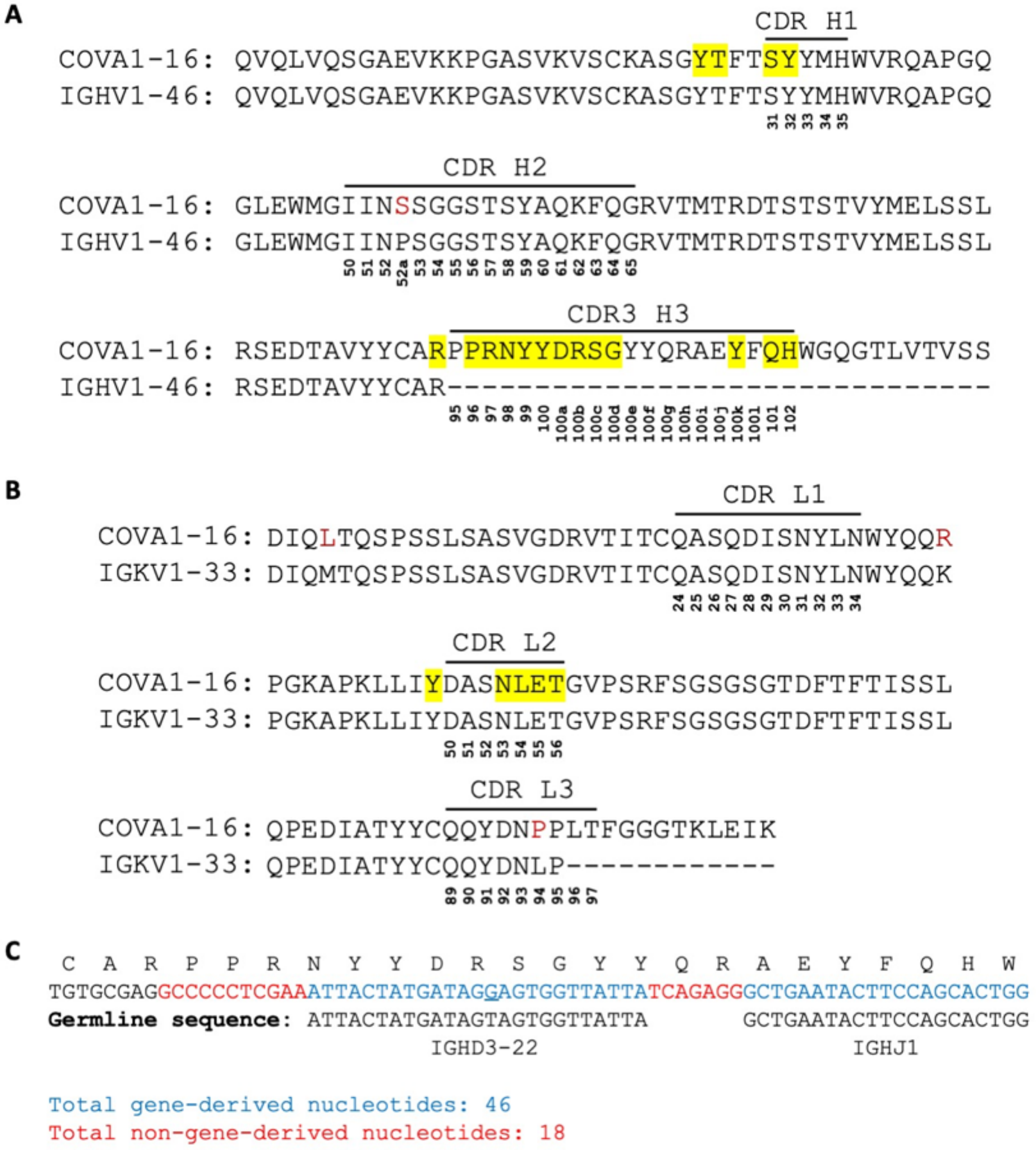

Figure S1, related to Figure 1. Comparison of COVA1-16 and putative germline sequences. Alignment of COVA1-16 Fab amino-acid sequence with **(A)** germline IGHV1-46 sequence, and **(B)** germline IGKV1-33 sequence. The regions that correspond to CDR H1, H2, H3, L1, L2, and L3 are indicated. Residues that differ from germline are highlighted in red. COVA1-16 Fab residues that interact with the RBD are highlighted in yellow [defined here as residues with a BSA > 0 Å^2 as calculated by the PISA program (Krissinel and Henrick, 2007)]. Residue positions in the CDRs are labeled according to the Kabat

numbering scheme. **(C)** Amino acid and nucleotide sequences of the V-D-J junction of COVA1-16, with putative gene segments (blue) and N-regions from N-addition (red), are indicated. The germline sequences of IGHD3-22 and IGHJ1 are also shown. The only somatically mutated nucleotide in the D region is underlined that results in a V_H S100bR mutation.

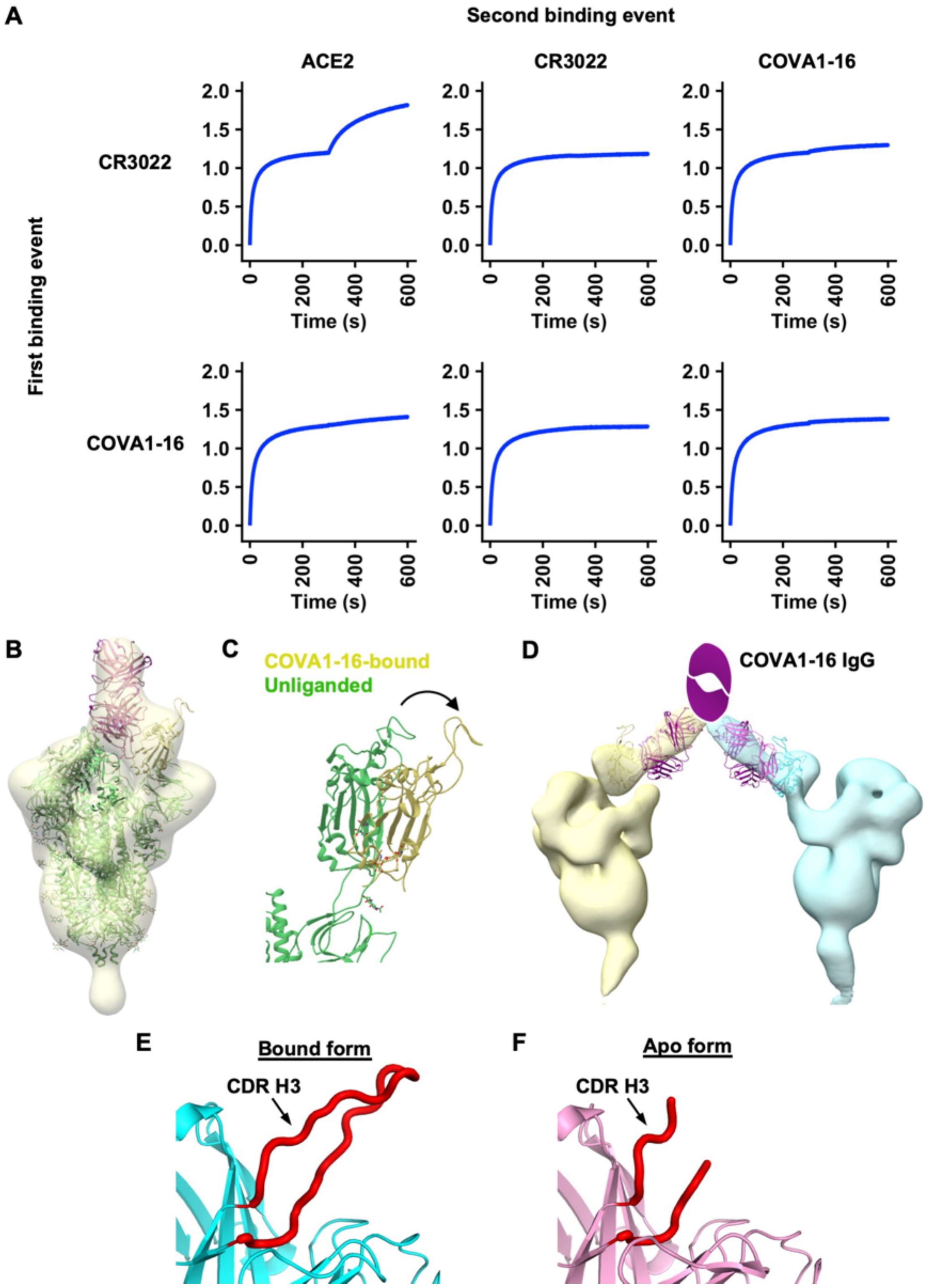

Figure S2, related to Figures 2 and 3. Competition assay between different IgGs and ACE2 and negative-stain EM analysis of COVA1-16 binding to SARS-CoV-2 S trimer.

Competition between COVA1-16 IgG, CR3022 IgG, and Fc-tagged ACE2 was measured by biolayer interferometry (BLI). Y-axis represents the response. The biosensor was first loaded with SARS-CoV-2 RBD, followed by two binding events: 1) CR3022 IgG or COVA1-16 IgG, and 2) ACE2, CR3022 IgG, or COVA1-16 IgG. A period of 300 s was used for each binding event. A further increase in signal during the second binding event (starting at 300 s time point) indicates lack of competition with the first ligand. **(B)** An atomic model from the crystal structure of SARS-CoV-2 RBD bound to COVA1-16 Fab was fit into the negative-stain EM reconstruction of the SARS-CoV-2 spike bound to COVA1-16 Fab. The COVA1-16 Fab approaches the apex of the S trimer in a perpendicular orientation. A secondary structure backbone representation of the prefusion spike model (PDB: 6Z97, green) (Huo et al., 2020) was also fit into the EM density with RBD residues (334-528) removed from one of the protomers here for clarity. The COVA1-16 heavy and light chains are in magenta and pink, respectively, and COVA1-16-bound RBD in yellow. **(C)** Conformation of RBD in an up conformation from an unliganded SARS-CoV-2 S trimer (PDB: 6Z97, green) (Huo et al., 2020) is compared to that of the RBD (yellow) bound by COVA1-16 Fab. The arrow indicates that the RBD further rotates and opens up when bound to COVA1-16, thereby moving further away from the trimer threefold axis. **(D)** An atomic model of the spike RBD bound to COVA1-16 Fab is fit into a negative-stain EM reconstruction, where COVA1-16 Fab approaches the SARS-CoV-2 S trimer from the side. COVA1-16 is modelled as an IgG to illustrate the feasibility of bivalent binding to adjacent spike proteins on the virus surface. The Fab heavy and light chains are shown in magenta and pink. A schematic representation of the Fc domain of the IgG is shown in magenta. The RBD model and spike density for each trimer is shown in yellow and cyan. **(E)** In the crystal structure of the RBD-bound form of COVA1-16 Fab, the CDR H3 loop is completely ordered (red). **(F)** In the crystal structure of the apo form of COVA1-16, the distal end of the CDR H3 loop is intrinsically disordered or flexible (red).

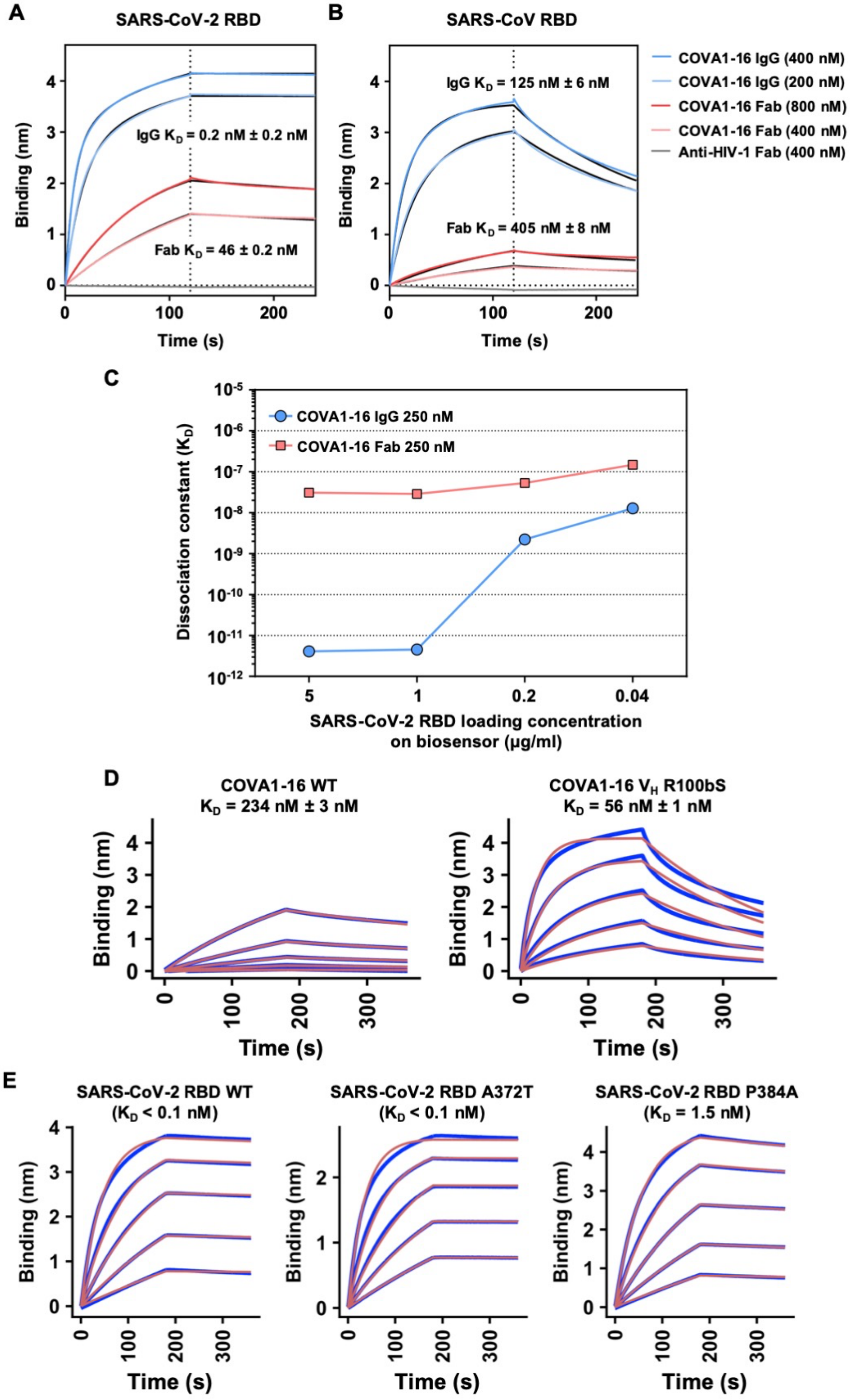

Figure S3, related to Figures 2 and 3. Sensorgrams for binding of COVA1-16 to SARS-CoV-2 RBD and SARS-CoV RBD. (A-B) Binding kinetics of COVA1-16 Fab and

IgG to **(A)** SARS-CoV-2 RBD and **(B)** SARS-CoV RBD were measured by biolayer interferometry (BLI) with RBD on the biosensor and antibody in solution. An anti-HIV His-tagged Fab (4E1) was used as a negative control. **(C)** The relationship between SARS-CoV-2 RBD loading concentration on the biosensor and the dissociation constant of COVA1-16 IgG is shown. **(D)** Binding kinetics of COVA1-16 wild-type and V_H R100bS mutant Fab to SARS-CoV-2 RBD were measured by biolayer interferometry (BLI) with RBD on the biosensor and antibody in solution. Unlike panels **A-C**, which used HEK293F-expressed SARS-CoV-2, the experiment here used insect cell-expressed SARS-CoV-2. **(E)** Binding kinetics of COVA1-16 IgG to SARS-CoV-2 RBD WT, A372T, and P384A were measured by biolayer interferometry (BLI) with RBD on the biosensor and antibody in solution. A372T and P384A are the only two mutations that differ between the SARS-CoV-2 and SARS-CoV sequences in COVA1-16 epitope. The affinity of COVA1-16 IgG to the A372T mutant did not show any detectable difference from WT. Although the affinity (K_D) of COVA1-16 IgG to the P384A mutant decreases, the binding is still 100 times tighter than that measured between COVA1-16 IgG and SARS-CoV RBD (see panel **B**). For all sensorgrams in this figure, Y-axis represents the response. Dissociation constants (K_D) for IgG and Fab were obtained using a 1:2 bivalent model and 1:1 binding model, respectively, which are represented by the red lines. Representative results of two replicates for each experiment are shown.

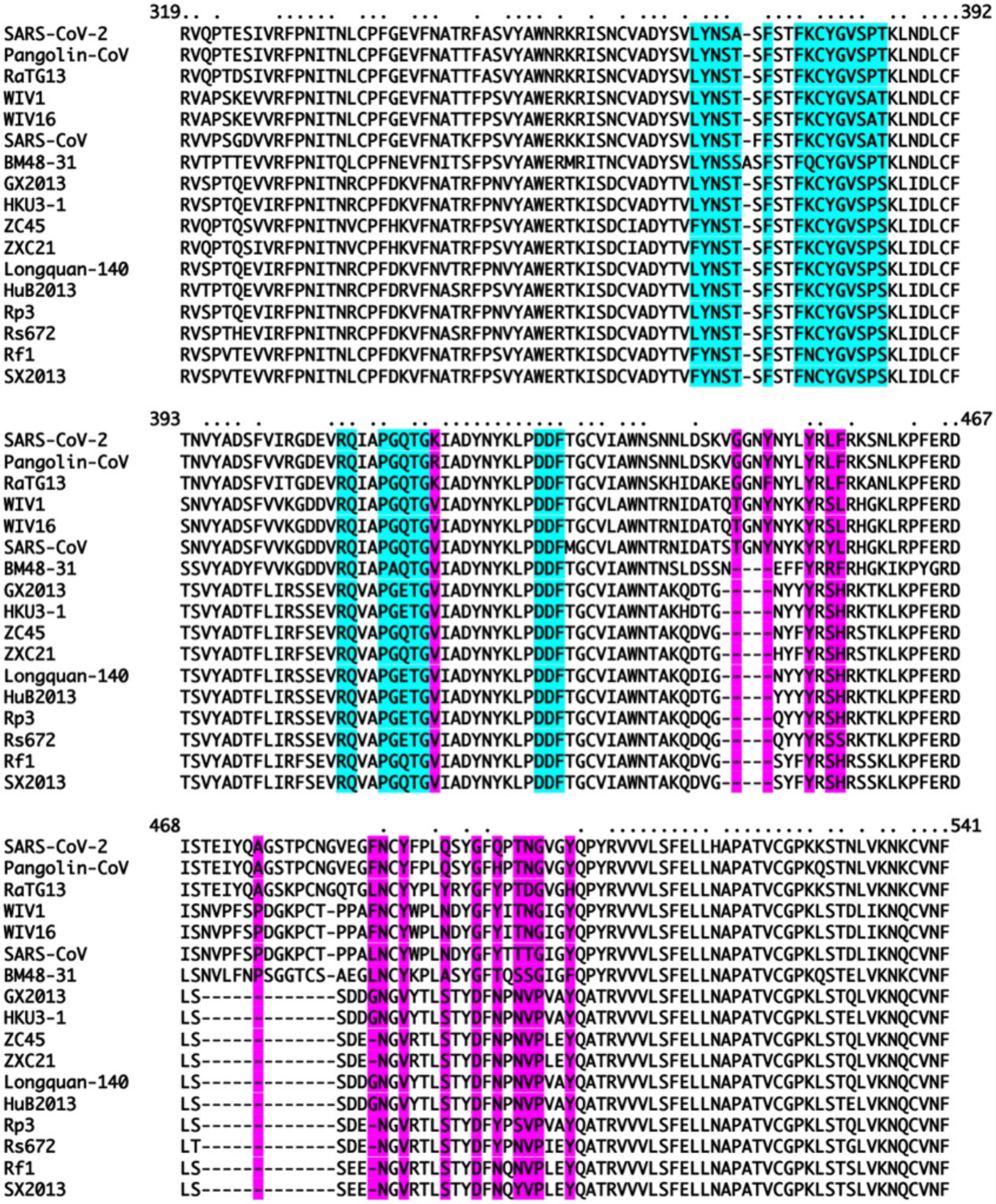

Figure S4, related to Figure 4. Sequence alignment of the RBD from SARS-related coronaviruses. Amino-acid sequences of RBDs from SARS-CoV-2, SARS-CoV, and other SARS-related coronavirus (SARSr-CoV) strains are aligned. COVA1-16 epitope residues are highlighted in cyan. ACE2-binding residues are highlighted in purple. Conserved residues are indicated by small black dots on the top of the alignment.

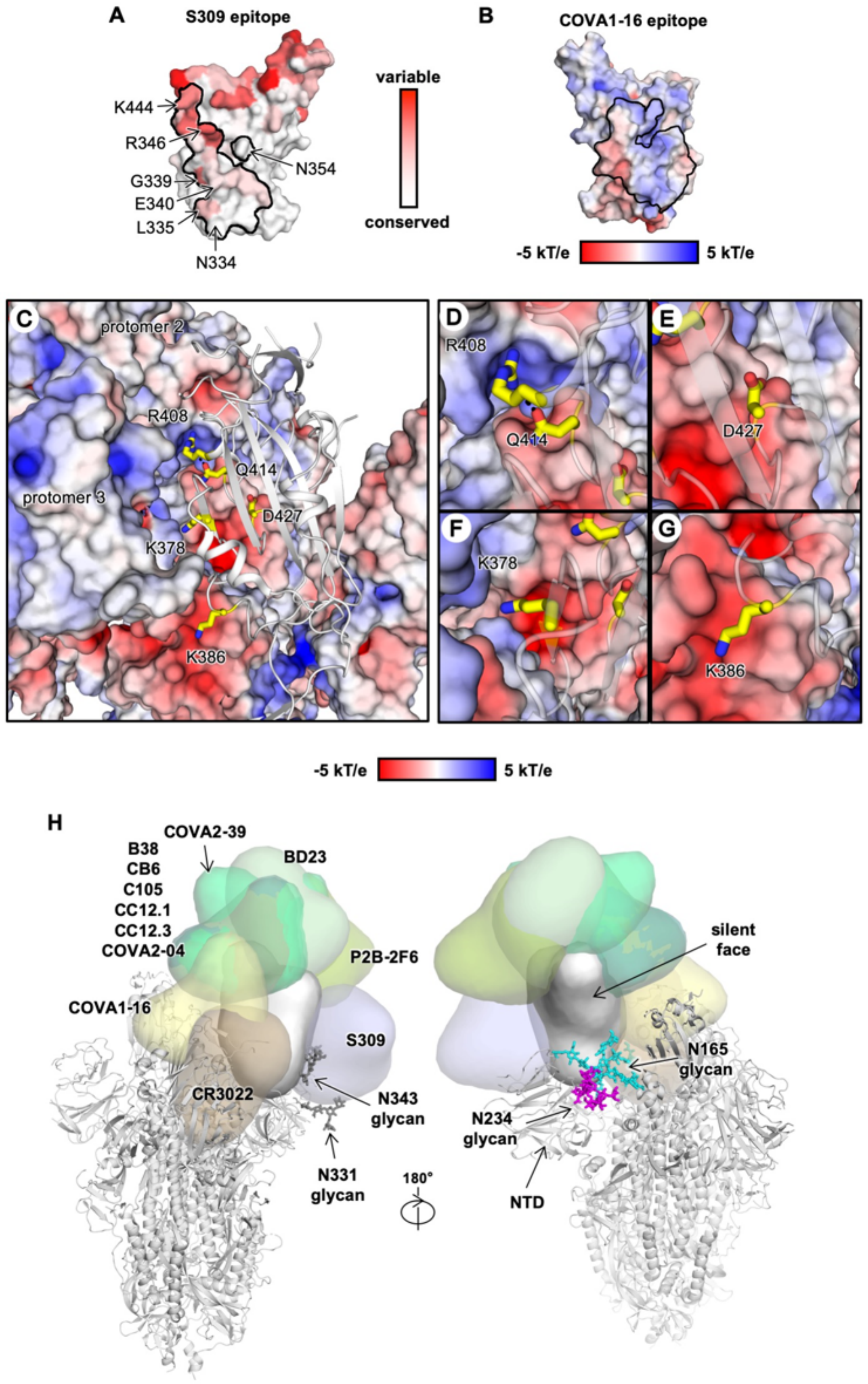

Figure S5, related to Figures 4 and 5. Sequence conservation of S309 epitope and additional structural analyses on COVA1-16 epitope. (A) Sequence conservation of

the RBD is highlighted on the structure for S309 epitope (Pinto et al., 2020). This view corresponds to the opposite side (rotated 180 degrees along the vertical axis) from that shown in Figure 4A-B. **(B)** The epitope of COVA1-16 is outlined and is mainly polar in character. **(C)** The RBD of one of the three protomers is shown as a gray cartoon with the side chains of five residues of interest shown in yellow stick representation. RBD residues K378, R408, Q414, and D427 are within the COVA1-16 epitope, whereas K386 is not a COVA1-16 epitope residue. The other two protomers (protomers 2 and 3) are shown in a surface electrostatic representation. **(D-G)** Zoomed-in views for the regions surrounding residues **(D)** R408 and Q414, **(E)** D427, **(F)** K378, and **(G)** K386. A hydrogen bond in **(D)** is represented by a dashed line. Due to charge difference or similarity between the side chain and the proximal region of the neighboring protomer, either repulsive (same charge) or attractive (opposite charge) environments are found and visualized here. PDB 6VXX is used to represent the spike protein (Walls et al., 2020). Of note, the shape complementarity values (Sc) (Lawrence and Colman, 1993) of the COVA1-16 epitope/RBD interface, COVA1-16 epitope/S2 interface, and COVA1-16 epitope/COVA1-16 interface are 0.53, 0.75, and 0.74, respectively, indicating good complementarity and tight fit of the COVA1-16 epitope surface with the rest of the trimer in the RBD down conformation. Sc values can range from 0 to 1, with a larger Sc value represents higher shape complementarity. **(H)** The antibody-bound RBD is shown in the up conformation on the S protein (PDB 6VSB) (Wrapp et al., 2020). N-glycans on N165 (NTD), N234, N331, and N343 (RBD) are modelled according to the main glycoform observed at these sites in (Watanabe et al., 2020) and shown in stick representation. Antibody Fabs from published crystal and cryo-EM structures are represented as globular outlines in different colors. B38, CB6, C105, CC12.1, CC12.3, COVA2-04, COVA2-39, BD23, P2B-2F6 all bind at or around the receptor binding site. S309 binds to the elongated accessible face of the RBD

in both up and down conformations, and CR3022 binds to the opposite face that is exposed in the RBD up conformation, but buried in the RBD down conformation.

Table S1, related to Figure 1. X-ray data collection and refinement statistics

**Data collection**		
	COVA1-16 Fab + SARS-CoV-2 RBD	COVA1-16 Fab
Beamline	SSRL 12-1	SSRL 12-1
Wavelength (Å)	0.97946	0.97946
Space group	$P\,1\,2_1\,1$	$P\,4_1\,3\,2$
Unit cell parameters		
a, b, c (Å)	57.4, 124.9, 57.6	156.3, 156.3, 156.3
α, β, γ (°)	90, 96.1, 90	90, 90, 90
Resolution (Å) [a]	50.0-2.89 (2.95-2.89)	50.0-2.53 (2.58-2.53)
Unique reflections [a]	17,656 (845)	22,357 (1,084)
Redundancy [a]	3.7 (3.2)	37.0 (14.1)
Completeness (%) [a]	97.9 (93.9)	100.0 (100.0)
$\langle I/\sigma_I \rangle$ [a]	7.4 (1.2)	21.5 (1.3)
R_{sym}[b] (%) [a]	15.3 (69.1)	23.6 (>100)
R_{pim}[b] (%) [a]	9.0 (42.9)	3.8 (54.3)
$CC_{1/2}$[c] (%) [a]	96.3 (66.8)	99.6 (52.1)
**Refinement statistics**		
Resolution (Å)	42.8-2.89	34.1-2.53
Reflections (work)	17,632	21,872
Reflections (test)	948	1,069
R_{cryst}[d] / R_{free}[e] (%)	23.7/29.4	21.2/24.4
No. of atoms	4,873	3,284
Macromolecules	4,845	3,223
Glycans	28	-
Average *B*-values ($Å^2$)	49	43
Macromolecules	49	43
Fab	45	43
RBD	56	-
Glycans	89	-
Wilson *B*-value ($Å^2$)	43	40
**RMSD from ideal geometry**		
Bond length (Å)	0.004	0.007
Bond angle (°)	0.74	1.02
**Ramachandran statistics (%)** [f]		
Favored	95.9	96.7
Outliers	0.16	0.0
**PDB code**	7JMW	7JMX

[a] Numbers in parentheses refer to the highest resolution shell.
[b] $R_{sym} = \Sigma_{hkl} \Sigma_i \mid I_{hkl,i} - \langle I_{hkl} \rangle \mid / \Sigma_{hkl} \Sigma_i I_{hkl,i}$ and $R_{pim} = \Sigma_{hkl} (1/(n-1))^{1/2} \Sigma_i \mid I_{hkl,i} - \langle I_{hkl} \rangle \mid / \Sigma_{hkl} \Sigma_i I_{hkl,i}$, where $I_{hkl,i}$ is the scaled intensity of the ith measurement of reflection h, k, l, $\langle I_{hkl} \rangle$ is the average intensity for that reflection, and *n* is the redundancy.
[c] $CC_{1/2}$ = Pearson correlation coefficient between two random half datasets.
[d] $R_{cryst} = \Sigma_{hkl} \mid F_o - F_c \mid / \Sigma_{hkl} \mid F_o \mid \times 100$, where F_o and F_c are the observed and calculated structure factors, respectively.
[e] R_{free} was calculated as for R_{cryst}, but on a test set comprising 5% of the data excluded from refinement.
[f] From MolProbity (Chen et al., 2010).

Table S2, related to Figure 1. Hydrogen bonds identified in the antibody-RBD interface using the PISA program

COVA1-16 Fab	Distance [Å]	SARS-CoV-2 RBD
H:ARG100b[NH2]	3.3	A:TYR369[O]
H:ARG100b[NE]	3.9	A:SER371[O]
H:ARG100b[N]	3.8	A:PHE377[O]
H:TYR100[N]	2.6	A:CYS379[O]
H:GLN101[NE2]	3.1	A:GLN414[OE1]
H:ARG97[NH1]	2.5	A:ASP427[O]
H:TYR32[OH]	3.1	A:ASP427[OD1]
H:THR28[N]	3.2	A:ASP427[OD2]
H:ARG97[NH1]	3.0	A:PHE429[O]
H:TYR100[O]	2.9	A:CYS379[N]
H:SER100c[O]	3.3	A:THR385[OG1]
H:GLN101[OE1]	3.8	A:GLN414[NE2]
L:ASN53[OD1]	3.2	A:ARG408[NH2]
L:LEU54[O]	3.7	A:ARG408[NE]
