# Supplementary material for: Cross-Neutralization of a SARS-CoV-2 Antibody to a Functionally Conserved Site Is Mediated by Avidity
Source: Immunity. 2020 Dec 15;53(6):1272–1280.e5. doi: 10.1016/j.immuni.2020.10.023 (PMC7687367; doi:10.1016/j.immuni.2020.10.023)
Supplement: Document S2. Article plus Supplemental Information [file mmc2.pdf]

Article

Immunity

Cross-Neutralization of a SARS-CoV-2 Antibody to a Functionally Conserved Site Is Mediated by Avidity

Graphical Abstract

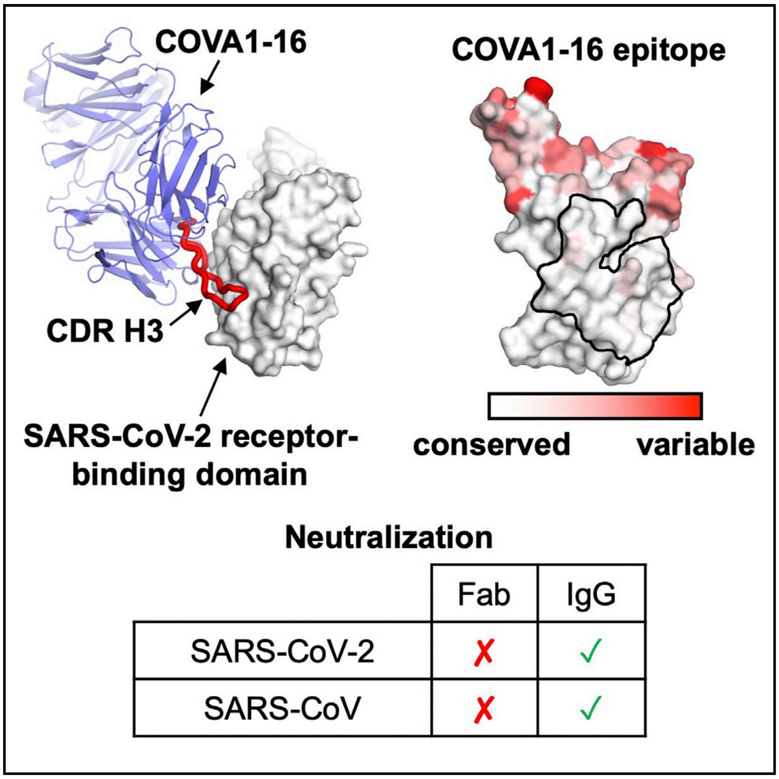

Authors

Hejun Liu, Nicholas C. Wu, Meng Yuan, ..., Rogier W. Sanders, Andrew B. Ward, Ian A. Wilson

Correspondence

wilson@scripps.edu

In Brief

COVA1-16 is a SARS-CoV-2 antibody from an individual with COVID-19 that cross-neutralizes SARS-CoV. Liu et al. reveal that COVA1-16 binds to a highly conserved epitope using a long CDR H3, where its approach angle sterically blocks ACE2 from engaging the RBS. Virus neutralization by COVA1-16 is driven by IgG avidity.

Highlights

- X-ray and EM structures of cross-neutralizing antibody COVA1-16 with SARS-CoV-2 RBD
- COVA1-16 binding to SARS-CoV-2 RBD is dominated by CDR H3
- COVA1-16 binds to a highly conserved non-RBS epitope but still competes with ACE2
- IgG avidity is the key for the cross-neutralization activity of COVA1-16

Liu et al., 2020, Immunity *53*, 1272–1280
December 15, 2020 © 2020 The Author(s). Published by Elsevier Inc.
https://doi.org/10.1016/j.immuni.2020.10.023

CellPress

Article

Cross-Neutralization of a SARS-CoV-2 Antibody to a Functionally Conserved Site Is Mediated by Avidity

Hejun Liu,[1,7] Nicholas C. Wu,[1,7] Meng Yuan,[1,7] Sandhya Bangaru,[1] Jonathan L. Torres,[1] Tom G. Caniels,[2] Jelle van Schooten,[2] Xueyong Zhu,[1] Chang-Chun D. Lee,[1] Philip J.M. Brouwer,[2] Marit J. van Gils,[2] Rogier W. Sanders,[2,3] Andrew B. Ward,[1,4,5] and Ian A. Wilson[1,4,5,6,8,*]

[1]Department of Integrative Structural and Computational Biology, The Scripps Research Institute, La Jolla, CA 92037, USA
[2]Department of Medical Microbiology, Amsterdam UMC, University of Amsterdam, Amsterdam, the Netherlands
[3]Department of Microbiology and Immunology, Weill Medical College of Cornell University, New York, NY 10021, USA
[4]IAVI Neutralizing Antibody Center, The Scripps Research Institute, La Jolla, CA 92037, USA
[5]Consortium for HIV/AIDS Vaccine Development (CHAVD), The Scripps Research Institute, La Jolla, CA 92037, USA
[6]The Skaggs Institute for Chemical Biology, The Scripps Research Institute, La Jolla, CA 92037, USA
[7]These authors contributed equally
[8]Lead Contact
*Correspondence: wilson@scripps.edu
https://doi.org/10.1016/j.immuni.2020.10.023

SUMMARY

Most antibodies isolated from individuals with coronavirus disease 2019 (COVID-19) are specific to severe acute respiratory syndrome coronavirus 2 (SARS-CoV-2). However, COVA1-16 is a relatively rare antibody that also cross-neutralizes SARS-CoV. Here, we determined a crystal structure of the COVA1-16 antibody fragment (Fab) with the SARS-CoV-2 receptor-binding domain (RBD) and negative-stain electron microscopy reconstructions with the spike glycoprotein trimer to elucidate the structural basis of its cross-reactivity. COVA1-16 binds a highly conserved epitope on the SARS-CoV-2 RBD, mainly through a long complementarity-determining region (CDR) H3, and competes with the angiotensin-converting enzyme 2 (ACE2) receptor because of steric hindrance rather than epitope overlap. COVA1-16 binds to a flexible up conformation of the RBD on the spike and relies on antibody avidity for neutralization. These findings, along with the structural and functional rationale for epitope conservation, provide insights for development of more universal SARS-like coronavirus vaccines and therapies.

INTRODUCTION

Human infection with severe acute respiratory syndrome coronavirus 2 (SARS-CoV-2) (Zhou et al., 2020b) rapidly escalated to an ongoing global pandemic of coronavirus disease 2019 (COVID-19) (Kissler et al., 2020). Given the current lack of protective vaccines and antiviral agents, virus clearance and recovery from SARS-CoV-2 have to rely mainly on generation of a neutralizing antibody response. To date, most neutralizing antibodies from convalescent individuals target the receptor-binding domain (RBD) on the trimeric spike (S) glycoprotein (Brouwer et al., 2020; Cao et al., 2020; Robbiani et al., 2020; Rogers et al., 2020; Zost et al., 2020), whose natural function is to mediate viral entry by first attaching to the human receptor angiotensin-converting enzyme 2 (ACE2) and then fusing its viral membrane with the host cell (Lan et al., 2020; Letko et al., 2020; Shang et al., 2020; Yan et al., 2020; Zhou et al., 2020b).

SARS-CoV-2 is phylogenetically closely related to SARS-CoV (Zhou et al., 2020b), which caused the 2002–2003 human epidemic. However, SARS-CoV-2 and SARS-CoV only share 73% amino acid sequence identity in their RBD compared with 90% in their S2 fusion domain. Nevertheless, a highly conserved epitope on the SARS-CoV-2 RBD has been identified previously from studies of a SARS-CoV-neutralizing antibody, CR3022 (Tian et al., 2020; Yuan et al., 2020b), which was originally isolated almost 15 years ago (ter Meulen et al., 2006). Many human monoclonal antibodies have now been shown to target the SARS-CoV-2 S protein (Andreano et al., 2020; Barnes et al., 2020; Brouwer et al., 2020; Cao et al., 2020; Chi et al., 2020; Ju et al., 2020; Li et al., 2020; Liu et al., 2020; Pinto et al., 2020; Robbiani et al., 2020; Rogers et al., 2020; Seydoux et al., 2020; Shi et al., 2020; Wec et al., 2020; Wu et al., 2020c; Yuan et al., 2020b; Zost et al., 2020), but cross-neutralizing antibodies are relatively uncommon in individuals with COVID-19 (Brouwer et al., 2020; Ju et al., 2020; Lv et al., 2020; Rogers et al., 2020). To date, the only structurally characterized cross-neutralizing human antibodies are S309 (Pinto et al., 2020) and ADI-56046 (Wec et al., 2020) from SARS-CoV survivors as well as EY6A from an individual with COVID-19 (Zhou et al., 2020a). Such structural and molecular characterization of cross-neutralizing antibodies is extremely valuable to understand how to confer broader protection against human SARS-like viruses that include the extensive reservoir of zoonotic sarbecoviruses in bats, pangolins, etc.

Here we determined the crystal structure of a cross-neutralizing human antibody, COVA1-16, in complex with the SARS-CoV-2 RBD. COVA1-16 utilizes a long complementarity-determining region (CDR) H3 to target a highly conserved epitope and can

 © 2020 The Author(s). Published by Elsevier Inc.
This is an open access article under the CC BY license (http://creativecommons.org/licenses/by/4.0/).

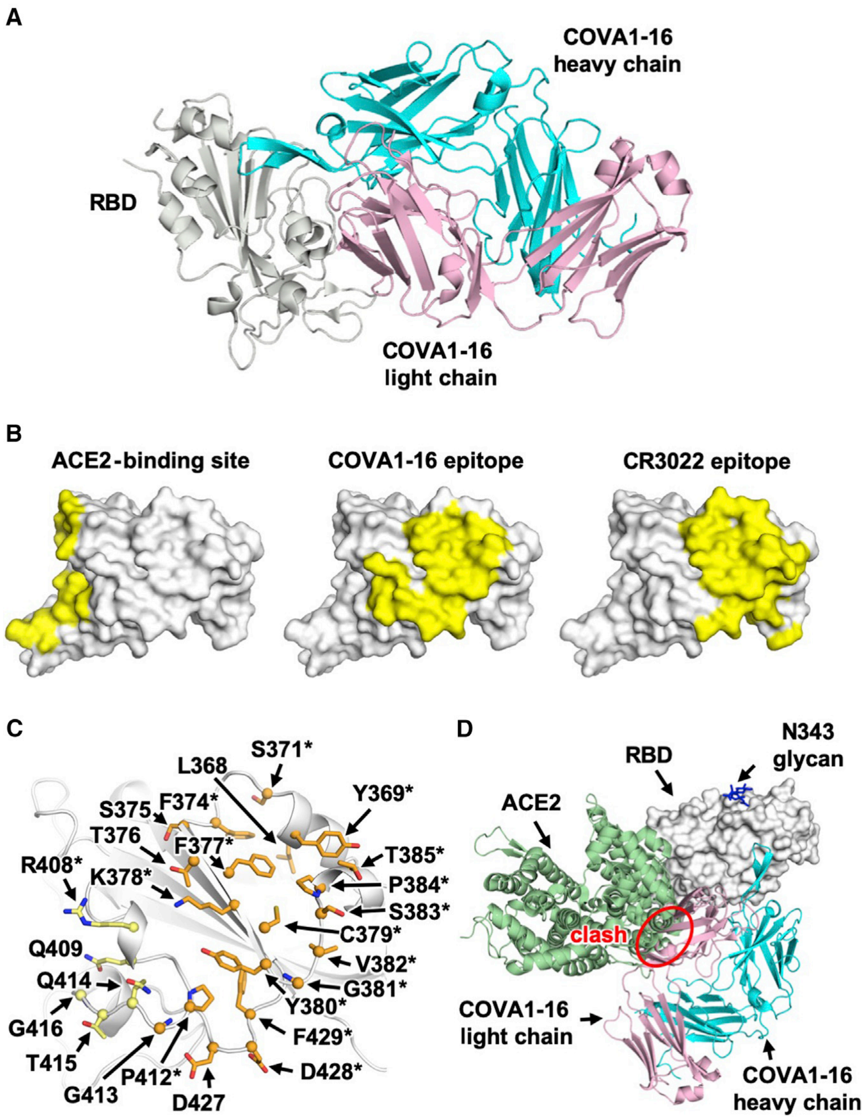

Figure 1. Comparison of the COVA1-16 Binding Mode with CR3022 and ACE2

(A) Crystal structure of the COVA1-16–RBD complex with the RBD in gray and the COVA1-16 Fab in cyan (heavy chain) and pink (light chain).

(B) The ACE2-binding site (PDB: 6M0J; left; Lan et al., 2020), the COVA1-16 epitope (this study, center), and the CR3022 epitope (PDB: 6W41; right; Yuan et al., 2020b) are highlighted in yellow.

(C) RBD residues in the COVA1-16 epitope. Epitope residues contacting the heavy chain are shown in orange and the light chain in yellow. Representative epitope residues are labeled. Residues that are also part of the CR3022 epitope are indicated by asterisks.

(D) The ACE2-RBD complex structure is aligned in the same orientation as the COVA1-16-RBD complex. The COVA1-16 light chain (pink) would clash with ACE2 (green) if they were to approach their respective RBD binding sites at the same time (indicated by a red circle).

See also Figure S1 and Tables S1 and S2.

cross-neutralize SARS-CoV. Although its epitope does not overlap with the ACE2 receptor binding site, COVA1-16 is able to compete with ACE2 for binding to the RBD. Our binding experiments and neutralization assays revealed that bivalent immunoglobulin G (IgG) binding is important for the neutralization activity of COVA1-16. We also performed a structural analysis to understand the functional constraints that underlie sequence conservation of the COVA1-16 epitope. This study provides insights into vaccine and therapy development for SARS-CoV-2 as well as other SARS-like viruses.

RESULTS

COVA1-16 Binds to a Conserved Epitope on the SARS-CoV-2 RBD that Overlaps with the CR3022 Epitope

The antibody COVA1-16 was recently isolated from an individual recovering from COVID-19 and cross-neutralizes SARS-CoV-2 (half-maximal inhibitory concentration [IC_{50}], 0.13 μg/mL) and SARS-CoV (IC_{50}, 2.5 μg/mL) pseudovirus (Brouwer et al., 2020). The heavy and light chains of COVA1-16 are encoded by immunoglobulin (IG) genes IGHV1-46, IGHD3-22, IGHJ1, IGKV1-33, and IGKJ4, with a relatively long CDR H3 of 20 amino acids (Figure S1). IGHV of COVA1-16 is only 1% somatically mutated in its nucleotide sequence (one amino acid change) from the germline gene, whereas its IGKV is 1.4% somatically mutated (three amino acid changes). Here we determined the crystal structure of COVA1-16 in complex with the SARS-CoV-2 RBD at 2.89-Å resolution to identify its binding site (epitope) and mechanism of cross-neutralization (Figure 1A; Table S1). The epitope of COVA1-16 overlaps extensively with that of CR3022 but also extends toward the periphery of the ACE2-binding site (Figure 1B; Yuan et al., 2020b). Seventeen of 25 residues in the COVA1-16 epitope overlap with the highly conserved CR3022 binding site (17 of 28 residues) (Figure 1C). Consistent with structural identification of its epitope, COVA1-16 can compete with CR3022 for RBD binding (Figure S2). COVA1-16 appears to have some resemblance to the SARS-CoV cross-neutralizing antibody ADI-56046, whose epitope appears to span the CR3022 epitope and ACE2-binding site, as indicated by negative-stain electron microscopy (nsEM) (Wec et al., 2020). COVA1-16 also competes with ACE2 for RBD binding (Figure S2; Brouwer et al., 2020), although its epitope does not overlap the ACE2-binding site (Figure 1B). Therefore, COVA1-16 inhibits ACE2 binding because of steric hindrance with its light chain rather than by direct interaction with the receptor binding site (Figure 1D).

The Neutralization Activity of COVA1-16 Is Promoted by IgG Bivalent Binding

The RBD can adopt up and down conformations on the S trimer (Ke et al., 2020; Wrapp et al., 2020b). Although the ACE2

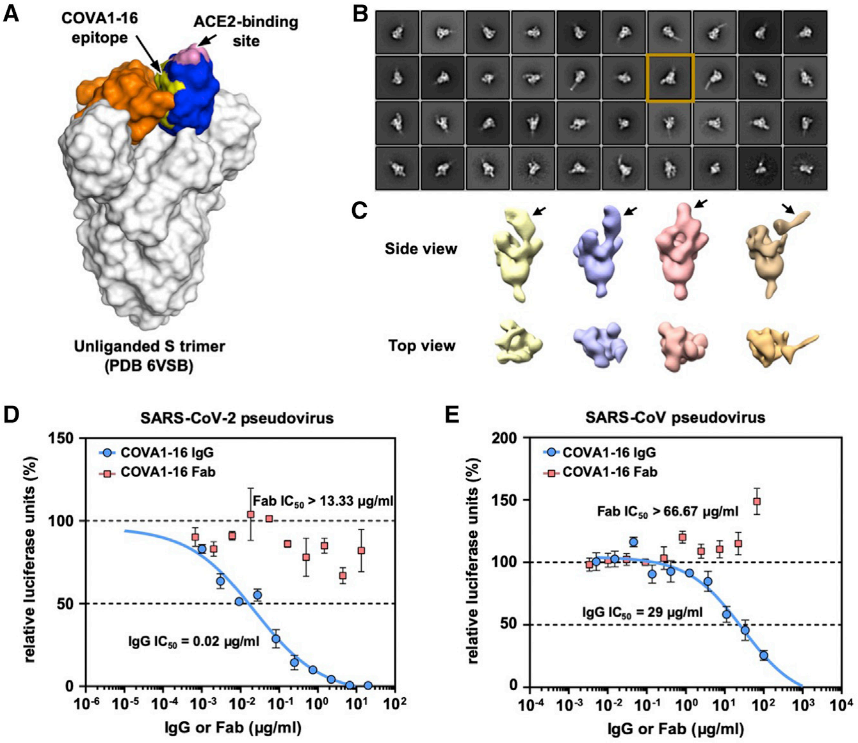

Figure 2. nsEM Analysis and IgG Avidity Effect of COVA1-16

(A) The COVA1-16 epitope on the unliganded SARS-CoV-2 S trimer with one RBD in the up conformation (blue) and two in the down conformation (orange) (PDB: 6VSB; Wrapp et al., 2020b). The COVA1-16 epitope is shown in yellow and the ACE2-binding site in pink.

(B) Representative 2D class averages derived from thousands of single-particle images from nsEM analysis of a SARS-CoV-2 S trimer complexed with the COVA1-16 Fab for a single experiment. The 2D class corresponding to the most outward conformation of the COVA-16 Fab in complex with the S trimer is highlighted in a mustard box.

(C) Various conformations of the COVA1-16 Fab in complex with the S trimer is revealed by 3D reconstruction. The location of the COVA1-16 Fab is indicated by an arrow.

(D and E) Neutralization activities of COVA1-16 IgG (blue) and the Fab (red) against (D) SARS-CoV-2 and (E) SARS-CoV are measured in a luciferase-based pseudovirus assay. The half-maximal inhibitory concentration (IC_{50}) values for IgG and the Fab are indicated in parentheses. Of note, neutralization of IgG (IC_{50} = 0.02 μg/mL) against the SARS-CoV-2 pseudovirus infecting 293T-ACE2 cells is comparable with that measured in Huh7 cells (IC_{50} = 0.13 μg/mL), as reported previously (Brouwer et al., 2020). Error bars indicate SEM of three technical replicates.

See also Figures S2 and S3.

receptor only binds the RBD in the up conformation (Yan et al., 2020), the previously characterized cross-neutralizing antibody S309 from an individual recovering from SARS-CoV and COVA2-15 from an individual with SARS-CoV-2 (Brouwer et al., 2020) can bind the RBD in the up and down conformations (Pinto et al., 2020; Wrapp et al., 2020b). However, unlike S309, the COVA1-16 epitope is completely buried when the RBD is in the down conformation (Figure 2A), akin to the CR3022 epitope (Yuan et al., 2020b). Even in the up conformation of the RBD on an unliganded SARS-CoV-2 S trimer (Wrapp et al., 2020b), the epitope of COVA1-16 would not be fully exposed (Figure 2A). We thus performed nsEM analysis of COVA1-16 in complex with the SARS-CoV-2 S trimer (Figure 2B). Three-dimensional (3D) reconstructions revealed that COVA1-16 can bind to a range of RBD orientations on the S protein when in the up position, indicating its rotational flexibility (Figure 2C). COVA1-16 could bind the S trimer from the top (i.e., perpendicular to the trimer apex; Figure 2C, yellow, blue, and pink) or from the side (i.e., more tilted; Figure 2C, brown). Model fitting of the COVA1-16-RBD crystal structure into the nsEM map indicated that the RBD on the S trimer is more open around the apex when COVA1-16 binds compared with unliganded trimers (Figures S2B and S2C). Bivalent binding of the COVA1-16 IgG between adjacent S trimers also appeared to be plausible (Figure S2D). A recent cryoelectron tomography (cryo-ET) analysis demonstrated that the average distance between prefusion S on the viral surface is around 150 Å (Yao et al., 2020), which is comparable with the distance between the tip of the two antibody fragments (Fabs) on an IgG (typically around 100–150 Å, although longer distances have been observed) (Klein and Bjorkman, 2010). Indeed, COVA1-16 IgG bound much more tightly than the Fab to the SARS-CoV-2 RBD, with dissociation constant (K_D) values of 0.2 nM and 46 nM, respectively (Figure S3A), reflecting bivalent binding in the assay format. Similarly, COVA1-16 IgG bound more strongly than the Fab to the SARS-CoV RBD (K_D of 125 nM versus 405 nM) (Figure S3B). Moreover, the apparent affinity of COVA1-16 IgG decreased to approximately the Fab value when the amount of SARS-CoV-2 RBD loaded on the biosensor was decreased, substantiating the notion that COVA1-16 can bind bivalently in this assay via interspike cross-linking (Figure S3C).

Bivalent IgG binding was also important for the neutralization activity of COVA1-16 (Figures 2D and 2E). COVA1-16 IgG neutralized SARS-CoV-2 pseudovirus with a half-maximal inhibitory concentration (IC_{50}) of 0.02 μg/mL, which is similar to that measured previously measured for SARS-CoV-2 pseudovirus (IC_{50} of 0.13 μg/mL) (Brouwer et al., 2020). In contrast, the COVA1-16 Fab did not neutralize SARS-CoV-2 pseudovirus even up to 13 μg/mL. A similar effect was also observed for SARS-CoV pseudovirus, which was neutralized by COVA1-16 IgG at an IC_{50} of 29 μg/mL but not by the COVA1-16 Fab even up to 67 μg/mL (Figure 2E). Of note, COVA1-16 is less potent against authentic SARS-CoV-2 (IC_{50} = 0.75 μg/mL) (Brouwer et al., 2020). Whether such a difference is due to variation in S protein density on the viral surface versus pseudovirus or due to other factors deserves future investigation. It will also be informative to compare the number, density, and conformational states of the S proteins on SARS-CoV-2 and SARS-CoV virions. Our findings support the importance of bivalent binding for SARS-CoV-2-neutralizing antibodies and especially

CellPress
OPEN ACCESS

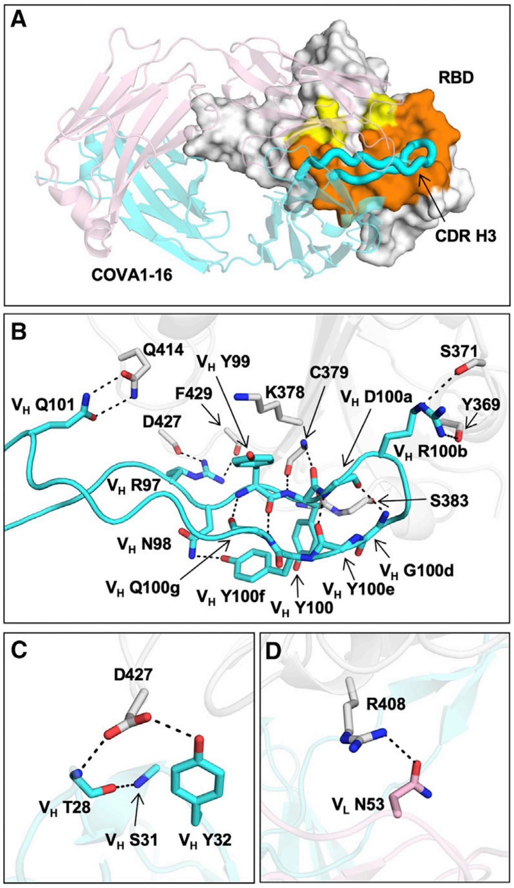

Figure 3. Interaction between the SARS-CoV-2 RBD and COVA1-16
(A) The epitope of COVA1-16 is highlighted in yellow and orange. Epitope residues that are in contact with CDR H3 are shown in orange and in yellow otherwise. COVA1-16 (heavy chain in cyan and light chain in pink) is in cartoon representation, with CDR H3 depicted as a thick tube. The RBD (white) is in a surface representation. The BSAs on COVA1-16 and RBD are 827 $Å^2$ and 780 $Å^2$, respectively.
(B–D) Interactions of the SARS-CoV-2 RBD (white) with (B) CDR H3 (cyan), (C) CDR H1 (cyan), and (D) CDR L2 (pink) of COVA1-16. H-bonds are represented by dashed lines. In (C), a 3_{10} turn is observed in CDR H1 for residues V_H T28–V_H S31.
See also Figures S2 and S3.

for cross-neutralization of SARS-CoV. Such a contribution of bivalent IgG (avidity) to SARS-CoV-2 neutralization has also been suggested in a recent study that compared binding of polyclonal IgGs and Fabs (Barnes et al., 2020). Furthermore, a single-domain camelid antibody, VHH-72, improved its neutralization activity to SARS-CoV-2 when expressed as a bivalent Fc fusion (Wrapp et al., 2020a). These observations are similar to some broadly neutralizing influenza antibodies to the hemagglutinin (HA) receptor binding site, where bivalent binding can increase avidity and neutralization breadth (Ekiert et al., 2012; Lee et al., 2012). Nevertheless, we have shown recently that the CR3022 Fab and IgG have similar neutralization potency as a SARS-CoV-2 variant with enhanced binding affinity for CR3022 (Wu et al., 2020a), suggesting that an avidity effect is not universally observed for all RBD-targeting antibodies, especially to this particular epitope, which is targeted by CR3022 and COVA1-16.

Binding of COVA1-16 to the RBD Is Dominated by CDR H3

Next we examined the molecular details of the interactions between COVA1-16 and SARS-CoV-2. COVA1-16 binding to the RBD is dominated by the heavy chain, which accounts for 81% of its total buried surface area (BSA; 673 $Å^2$ of a total of 827 $Å^2$). Most of the interactions are mediated by CDR H3 (Figure 3A), which contributes 72% (594 $Å^2$) of the total BSA. CDR H3 forms a beta-hairpin with a type I beta-turn at its tip and is largely encoded by IGHD3-22 (from N98 to heavy chain variable domain (V_H) Y100f; Figure S1C; Figure 3B). The beta-hairpin conformation is stabilized by four main chain-main chain hydrogen bonds (H-bonds) and a side chain-side chain H-bond between V_H N98 and V_H Y100f at either end of the IGHD3-22-encoded region (Figure 3B). Four H-bonds between the tip of CDR H3 and the RBD are formed from two main chain-main chain interactions with RBD C379 and two with V_H R100b (Table S2). The positively charged guanidinium of V_H R100b also interacts with the partial negative dipole at the C terminus of a short α helix in the RBD (residues Y365–Y369). V_H R100b is a somatically mutated residue (codon = AGG in the IGHD3-22-encoded region, where the germline residue is a Ser [codon = AGT]; Figure S1C). The short Ser side chain would likely not contact the RBD or provide electrostatic complementarity. A somatic revertant V_H R100bS actually improved the binding affinity of COVA1-16 to the RBD, mostly because of an increased on rate (Figure S3D). Nevertheless, COVA1-16 has a much slower off rate than its V_H R100bS mutant, which may have led to its selection. The CDR H3 tip also interacts with the RBD through hydrophobic interactions between V_H Y99 and the aliphatic portion of RBD K378 as well as a π-π interaction between V_H Y100 and the RBD V382-S383 peptide backbone (Figure 3B). CDR H3 forms an additional four H-bonds with the RBD, involving the side chains of V_H R97 and Q101 (Figure 3B). We further determined the unliganded structure of COVA1-16 Fab to 2.53-Å resolution and found that the CDR H3 distal region was not resolved because of lack of electron density, indicating its inherent flexibility (Figures S2E and S2F). CDR H1 and CDR L2 of COVA1-16 also interact with the RBD, but much less so compared with CDR H3. The V_H T28 main chain and V_H Y32 side chain in CDR H1 H-bond with D427 (Figure 3C; Table S2), whereas V_L N53 in CDR L2 H-bonds with RBD R408 (Figure 3D; Table S2).

Although SARS-CoV-2 and SARS-CoV differ by only two amino acid residues (A372T and P384A) in the COVA1-16 epitope (Figure S4), they do not appear to account for the affinity differences in COVA1-16 binding to the RBD (Figure S3E). As a result, the binding affinity of COVA1-16 to the RBD may be influenced by residues outside of the epitope as well as the dynamics of the RBD fluctuations between up and down conformations.

Sequence Conservation of the COVA1-16 Epitope Appears to Arise from Functional Constraints in the S Protein

Compared with the ACE2-binding site, the COVA1-16 epitope is much more highly conserved among SARS-CoV-2, SARS-CoV, and other SARS-related coronaviruses (SARSr-CoVs) (Figures 4A–4D; Figures S4 and S5A; Brouwer et al., 2020). Consistent with the sequence conservation of the epitope, COVA1-16 could bind to RBDs from Guangdong pangolin CoV and bat CoV

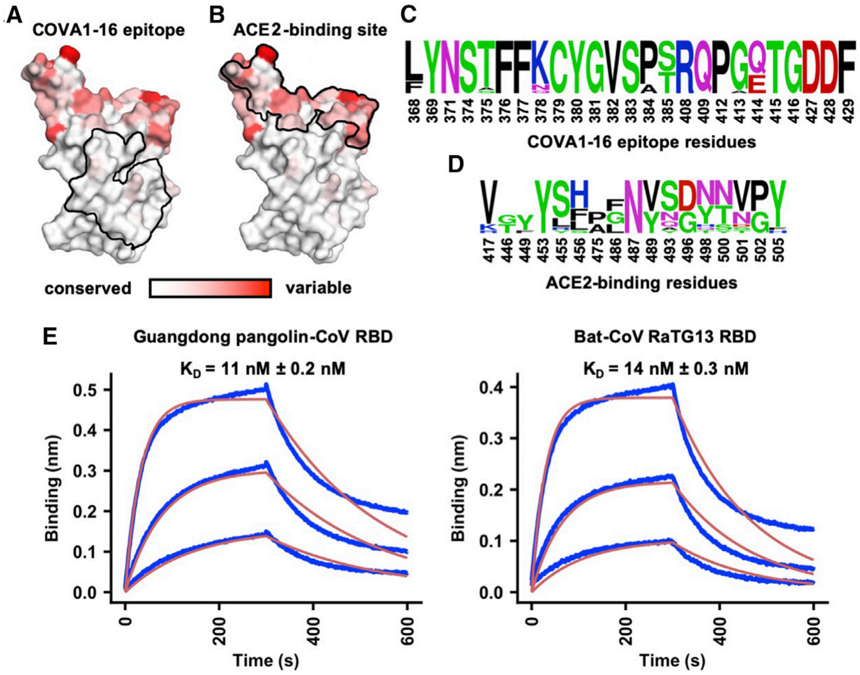

Figure 4. Sequence Conservation of the COVA1-16 Epitope and ACE2-Binding Site

(A and B) Sequence conservation of the RBD among 17 SARS-like CoVs (Figure S4) is highlighted on the RBD structure, with (A) COVA1-16 epitope and (B) ACE2-binding site indicated by the black outline. The backside of this view is shown in Figure S5A.

(C and D) Sequence conservation of the (C) COVA1-16 epitope and (D) ACE2-binding site is shown as a sequence logo.

(E) The binding kinetics of COVA1-16 IgG to RBDs from Guangdong pangolin CoV and bat CoV RaTG13 were measured by biolayer interferometry (BLI) with IgG on the biosensor and RBD in solution. The y axis represents the response. Dissociation constant (K_D) values were obtained using a 1:1 binding model and are represented by the red lines. Representative results of two replicates for each experiment are shown.

See also Figures S4 and S5.

RaTG13 (Figure 4E). To investigate possible structural and functional reasons for this sequence conservation, we analyzed the epitope location in the context of the SARS-CoV-2 trimeric S protein with all RBDs in the down conformation (Walls et al., 2020; Figure 5A; Figure S4). The COVA1-16 epitope is completely buried at the center of the trimer in the interface between the S1 and S2 domains and is largely hydrophilic (Figure S5B). The polar side chains of K378, Q414, R408, and D427, which are involved in binding to COVA1-16, are all very close to the interface with adjacent protomers in the S trimer. The R408 side chain, which is positioned by Q414 via an H-bond, points toward a region in the adjacent protomer 2 with a positive electrostatic potential. Similarly, D427 is juxtaposed to a region in protomer 2 with a negative electrostatic potential. These repulsive charges would help favor the metastability required for transient opening and closing of the RBD in up and down conformations prior to ACE2 receptor binding. In contrast, the K378 side chain points toward a region in protomer 3 with negative electrostatic potential, favoring the down RBD conformation. Furthermore, in the down conformation, part of the COVA1-16 epitope interacts with the long helices formed by the heptad repeat motifs of the S2 fusion domain (Figures 5A and 5B). Notably, S383 and T385 in the COVA1-16 epitope make three H-bonds with the tops of the helices and their connecting regions (Figure 5B). This mixture of attractive and repulsive forces would seem to be important for control of the dynamics of the RBD and, hence, for the biological function of the metastable pre-fusion S protein in receptor binding and fusion. The complementarity of fit of the epitope interface with the other RBDs and the S2 domain in the S trimer further explains the epitope conservation (Figures S5C–S5G). Therefore, the high sequence conservation of the COVA1-16 epitope appears to be related to the functional requirement for this component of the RBD surface to be deeply buried within the S trimer in the down conformation. The COVA1-16 epitope and its interaction with the RBD on the S compared with other antibody epitopes is illustrated in Figure S5H. Only a small part of the RBD surface ("silent face") has not yet been observed to bind antibodies to SARS-CoV-2.

DISCUSSION

From the SARS-CoV-2 RBD-antibody complex structures to date, a substantial portion of the RBD surface can be targeted by antibodies (Yuan et al., 2020c). One surface not yet observed to be targeted is partially covered by N-glycans at residues N165 on the N-terminal domain (NTD) and N343 on the RBD (Watanabe et al., 2020), which may hinder B cell receptor access and create a silent face, although the N343 glycan is incorporated in the S309 epitope (Pinto et al., 2020). While antibodies that target the ACE2-binding site, such as BD23 (Cao et al., 2020), CB6 (Shi et al., 2020), B38 (Wu et al., 2020c), P2B-2F6 (Ju et al., 2020), CC12.1 (Yuan et al., 2020a), CC12.3 (Yuan et al., 2020a), COVA2-04 (Wu et al., 2020b), and COVA2-39 (Wu et al., 2020b), do not show cross-neutralization activity to SARS-CoV, conserved epitopes that seem to be more able to support cross-neutralization can be found elsewhere (Pinto et al., 2020; Yuan et al., 2020b; Zhou et al., 2020a). So far, these rare cross-neutralizing antibodies, including COVA1-16, often seem to bind to epitopes that are not readily accessible in the pre-fusion native structure when the RBD is in the down conformation (Wec et al., 2020; Zhou et al., 2020a). This finding is similar to a recent discovery in influenza virus, where a class of cross-protective antibodies target a conserved epitope in the trimeric interface of the HA (Bajic et al., 2019; Bangaru et al., 2019; Watanabe et al., 2019). Because of the inaccessibility of the COVA1-16 epitope on the S protein, it is possible that an RBD-based rather than an S-based immunogen can elicit larger numbers of COVA1-16-like antibodies.

A main feature of COVA1-16 is its CDR H3-dominant binding mode. In fact, CDR H3-dominant antibodies have been seen in

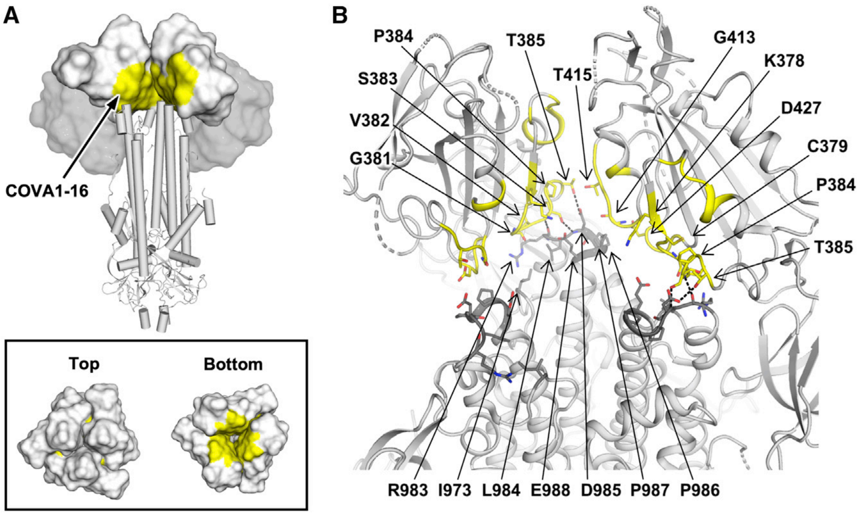

Figure 5. Structural and Functional Constraints of the COVA1-16 Epitope

(A) Location of the COVA1-16 epitope (yellow) on the SARS-CoV-2 S trimer when all three RBDs are in the down conformation (PDB: 6VXX; Walls et al., 2020). RBDs are represented as a white surface, N-terminal domains (NTDs) as a gray surface, and the S2 domain in a cartoon representation. Top panel: for visualization of the COVA1-16 epitope, the RBD and NTD from one of the three protomers was removed. Bottom panel: top and bottom views of the COVA1-16 epitopes on the three RBDs in the down conformation.

(B) The COVA1-16 epitope is shown in yellow on a ribbon representation of a SARS-CoV-2 S trimer (PDB: 6VXX; Walls et al., 2020). Epitope residues in the RBD involved in interaction with the S2 domain are shown as yellow sticks and S2 domain-interacting residues as dark gray sticks. Dashed lines indicate H-bonds. Interface residues were calculated using PISA software (Krissinel and Henrick, 2007). The S1 segment from the third protomer is omitted to clarify the view of the interfaces the COVA1-16 epitope makes with the S2 domain.

See also Figure S5.

the human immune response to other viral pathogens. Some pertinent examples are the antibodies PG9 and PG16, whose CDR H3s interact extensively along their length with the apex of the HIV-1 Envelope protein (McLellan et al., 2011; Pan et al., 2020). Another example is C05, which is essentially a single loop binder that inserts its very long CDR H3 (24 residues) into the RBD of influenza HA (Ekiert et al., 2012), providing a template for design of a high-avidity protein inhibitor of influenza virus, where the H3 loop is fused to a scaffold protein (Strauch et al., 2017). The long CDR H3 of COVA1-16 may similarly facilitate therapeutic designs that could also include peptide-based antiviral agents, as exemplified by a potent cyclic peptide fusion inhibitor of influenza HA (Corti et al., 2011; Kadam et al., 2017).

As SARS-CoV-2 continues to circulate in the human population, and other zoonotic coronaviruses constitute future pandemic threats (Menachery et al., 2015), it is certainly worth considering development of more universal coronavirus vaccines and therapeutic agents that can cross-neutralize antigenically drifted SARS-CoV-2 viruses as well as zoonotic SARS-like coronaviruses. This process will highly benefit from continued characterization of cross-neutralizing antibodies, as demonstrated for influenza virus (Wu and Wilson, 2018) and HIV (Ward and Wilson, 2020).

STAR★METHODS

Detailed methods are provided in the online version of this paper and include the following:

- KEY RESOURCES TABLE
- RESOURCE AVAILABILITY
  - Lead Contact
  - Materials Availability
  - Data and Code Availability
- EXPERIMENTAL MODEL AND SUBJECT DETAILS
  - Expression and Purification of SARS-CoV, SARS-CoV-2, and SARSr-CoV RBDs
  - Expression and Purification of COVA1-16 Fab
  - Expression and Purification of ACE2
  - Crystallization and X-ray Structure Determination
  - Expression and Purification of Recombinant S Protein for Negative-Stain EM
  - nsEM Sample Preparation and Data Collection
  - Protein Expression and Purification for Antibody Binding Studies
  - Measurement of Binding Affinities Using Biolayer Interferometry
  - Competition Studies of Antibodies with ACE2 Receptor
  - Pseudovirus Neutralization Assay
  - Shape Complementarity Analysis
  - Sequence Conservation Analysis
- QUANTIFICATION AND STATISTICAL ANALYSIS

SUPPLEMENTAL INFORMATION

Supplemental Information can be found online at https://doi.org/10.1016/j.immuni.2020.10.023.

ACKNOWLEDGMENTS

We thank Henry Tien for technical support with the crystallization robot, Jeanne Matteson and Yuanzi Hua for contributions to mammalian cell culture, Wenli Yu for insect cell culture, Robyn Stanfield for assistance with data collection, and Paul Bieniasz for cells and plasmids for the pseudovirus neutralization assays. We are grateful to the staff of Stanford Synchrotron Radiation Laboratory (SSRL) Beamline 12-1 for assistance. This work was supported by NIH K99 AI139445 (to N.C.W.), the Bill and Melinda Gates Foundation (OPP1170236 to A.B.W. and I.A.W. and OPP1132237 and INV-002022 to R.W.S.), NIH HIVRAD (P01 AI110657 to R.W.S., A.B.W., and I.A.W.), NIH CHAVD (UM1 AI44462 to A.B.W. and I.A.W.), a Netherlands Organisation for Scientific Research (NWO) Vici grant (to R.W.S.), the Fondation Dormeur, Vaduz (to M.J.v.G.), and a Health-Holland PPS allowance (LSHM20040 to

M.J.v.G.). M.J.v.G. is a recipient of an AMC fellowship and a COVID-19 grant from the Amsterdam Institute of Infection and Immunity. J.v.S. is a recipient of a 2017 AMC PhD scholarship. Use of the SSRL, SLAC National Accelerator Laboratory, is supported by the U.S. Department of Energy, Office of Science, Office of Basic Energy Sciences under contract DE-AC02–76SF00515. The SSRL Structural Molecular Biology Program is supported by the DOE Office of Biological and Environmental Research and the National Institutes of Health, National Institute of General Medical Sciences (including P41GM103393).

AUTHOR CONTRIBUTIONS

H.L., N.C.W., M.Y., and I.A.W. conceived and designed the study. H.L., N.C.W., M.Y., and C.-C.D.L. expressed and purified the proteins for crystallization. T.G.C., P.J.M.B., M.J.v.G., and R.W.S. provided antibody clones and sequences. T.G.C. performed binding analyses, and J.v.S. provided neutralization data. H.L., N.C.W., M.Y., and X.Z. crystallized and determined the X-ray structures. S.B., J.L.T., and A.B.W. provided nsEM data and reconstruction. H.L., N.C.W., M.Y., and I.A.W. wrote the paper, and all authors reviewed and/or edited the paper.

DECLARATION OF INTERESTS

A patent application for the SARS-CoV-2 antibody COVA1-16 and other antibodies first disclosed by Brouwer et al. (2020) has been filed by Amsterdam UMC under application number 2020-039EP-PR. I.A.W. is a member of the Immunity Editorial Board.

Received: August 21, 2020
Revised: September 27, 2020
Accepted: October 28, 2020
Published: November 25, 2020

REFERENCES

Adams, P.D., Afonine, P.V., Bunkóczi, G., Chen, V.B., Davis, I.W., Echols, N., Headd, J.J., Hung, L.W., Kapral, G.J., Grosse-Kunstleve, R.W., et al. (2010). PHENIX: a comprehensive Python-based system for macromolecular structure solution. Acta Crystallogr. D Biol. Crystallogr. *66*, 213–221.

Andreano, E., Nicastri, E., Paciello, I., Pileri, P., Manganaro, N., Piccini, G., Manenti, A., Pantano, E., Kabanova, A., Troisi, M., et al. (2020). Identification of neutralizing human monoclonal antibodies from Italian Covid-19 convalescent patients. bioRxiv. https://doi.org/10.1101/2020.05.05.078154.

Ashkenazy, H., Abadi, S., Martz, E., Chay, O., Mayrose, I., Pupko, T., and Ben-Tal, N. (2016). ConSurf 2016: an improved methodology to estimate and visualize evolutionary conservation in macromolecules. Nucleic Acids Res. *44* (W1), W344-50.

Baden, E.M., Owen, B.A., Peterson, F.C., Volkman, B.F., Ramirez-Alvarado, M., and Thompson, J.R. (2008). Altered dimer interface decreases stability in an amyloidogenic protein. J. Biol. Chem. *283*, 15853–15860.

Bajic, G., Maron, M.J., Adachi, Y., Onodera, T., McCarthy, K.R., McGee, C.E., Sempowski, G.D., Takahashi, Y., Kelsoe, G., Kuraoka, M., and Schmidt, A.G. (2019). Influenza antigen engineering focuses immune responses to a subdominant but broadly protective viral epitope. Cell Host Microbe *25*, 827–835.e6.

Bangaru, S., Lang, S., Schotsaert, M., Vanderven, H.A., Zhu, X., Kose, N., Bombardi, R., Finn, J.A., Kent, S.J., Gilchuk, P., et al. (2019). A site of vulnerability on the influenza virus hemagglutinin head domain trimer interface. Cell *177*, 1136–1152.e18.

Barnes, C.O., West, A.P., Jr., Huey-Tubman, K.E., Hoffmann, M.A.G., Sharaf, N.G., Hoffman, P.R., Koranda, N., Gristick, H.B., Gaebler, C., Muecksch, F., et al. (2020). Structures of human antibodies bound to SARS-CoV-2 spike reveal common epitopes and recurrent features of antibodies. Cell *182*, 828–842.e16.

Brouwer, P.J.M., Caniels, T.G., van der Straten, K., Snitselaar, J.L., Aldon, Y., Bangaru, S., Torres, J.L., Okba, N.M.A., Claireaux, M., Kerster, G., et al. (2020). Potent neutralizing antibodies from COVID-19 patients define multiple targets of vulnerability. Science *369*, 643–650.

Cao, Y., Su, B., Guo, X., Sun, W., Deng, Y., Bao, L., Zhu, Q., Zhang, X., Zheng, Y., Geng, C., et al. (2020). Potent neutralizing antibodies against SARS-CoV-2 identified by high-throughput single-cell sequencing of convalescent patients' B cells. Cell *182*, 73–84.e16.

Chen, V.B., Arendall, W.B., 3rd, Headd, J.J., Keedy, D.A., Immormino, R.M., Kapral, G.J., Murray, L.W., Richardson, J.S., and Richardson, D.C. (2010). MolProbity: all-atom structure validation for macromolecular crystallography. Acta Crystallogr. D Biol. Crystallogr. *66*, 12–21.

Chi, X., Yan, R., Zhang, J., Zhang, G., Zhang, Y., Hao, M., Zhang, Z., Fan, P., Dong, Y., Yang, Y., et al. (2020). A neutralizing human antibody binds to the N-terminal domain of the Spike protein of SARS-CoV-2. Science *369*, 650–655.

Corti, D., Voss, J., Gamblin, S.J., Codoni, G., Macagno, A., Jarrossay, D., Vachieri, S.G., Pinna, D., Minola, A., Vanzetta, F., et al. (2011). A neutralizing antibody selected from plasma cells that binds to group 1 and group 2 influenza A hemagglutinins. Science *333*, 850–856.

Crooks, G.E., Hon, G., Chandonia, J.M., and Brenner, S.E. (2004). WebLogo: a sequence logo generator. Genome Res. *14*, 1188–1190.

Edgar, R.C. (2004). MUSCLE: multiple sequence alignment with high accuracy and high throughput. Nucleic Acids Res. *32*, 1792–1797.

Ekiert, D.C., Friesen, R.H., Bhabha, G., Kwaks, T., Jongeneelen, M., Yu, W., Ophorst, C., Cox, F., Korse, H.J., Brandenburg, B., et al. (2011). A highly conserved neutralizing epitope on group 2 influenza A viruses. Science *333*, 843–850.

Ekiert, D.C., Kashyap, A.K., Steel, J., Rubrum, A., Bhabha, G., Khayat, R., Lee, J.H., Dillon, M.A., O'Neil, R.E., Faynboym, A.M., et al. (2012). Cross-neutralization of influenza A viruses mediated by a single antibody loop. Nature *489*, 526–532.

Emsley, P., Lohkamp, B., Scott, W.G., and Cowtan, K. (2010). Features and development of Coot. Acta Crystallogr. D Biol. Crystallogr. *66*, 486–501.

Fenn, S., Schiller, C.B., Griese, J.J., Duerr, H., Imhof-Jung, S., Gassner, C., Moelleken, J., Regula, J.T., Schaefer, W., Thomas, M., et al. (2013). Crystal structure of an anti-Ang2 CrossFab demonstrates complete structural and functional integrity of the variable domain. PLoS ONE *8*, e61953.

Huo, J., Zhao, Y., Ren, J., Zhou, D., Duyvesteyn, H.M.E., Ginn, H.M., Carrique, L., Malinauskas, T., Ruza, R.R., Shah, P.N.M., et al. (2020). Neutralization of SARS-CoV-2 by destruction of the prefusion spike. Cell Host Microbe *28*, 445–454.e6.

Ju, B., Zhang, Q., Ge, J., Wang, R., Sun, J., Ge, X., Yu, J., Shan, S., Zhou, B., Song, S., et al. (2020). Human neutralizing antibodies elicited by SARS-CoV-2 infection. Nature *584*, 115–119.

Kadam, R.U., Juraszek, J., Brandenburg, B., Buyck, C., Schepens, W.B.G., Kesteleyn, B., Stoops, B., Vreeken, R.J., Vermond, J., Goutier, W., et al. (2017). Potent peptidic fusion inhibitors of influenza virus. Science *358*, 496–502.

Ke, Z., Oton, J., Qu, K., Cortese, M., Zila, V., McKeane, L., Nakane, T., Zivanov, J., Neufeldt, C.J., Cerikan, B., et al. (2020). Structures and distributions of SARS-CoV-2 spike proteins on intact virions. Nature. Published online August 17, 2020. https://doi.org/10.1038/s41586-020-2665-2.

Kissler, S.M., Tedijanto, C., Goldstein, E., Grad, Y.H., and Lipsitch, M. (2020). Projecting the transmission dynamics of SARS-CoV-2 through the postpandemic period. Science *368*, 860–868.

Klein, J.S., and Bjorkman, P.J. (2010). Few and far between: how HIV may be evading antibody avidity. PLoS Pathog. *6*, e1000908.

Krissinel, E., and Henrick, K. (2007). Inference of macromolecular assemblies from crystalline state. J. Mol. Biol. *372*, 774–797.

Lan, J., Ge, J., Yu, J., Shan, S., Zhou, H., Fan, S., Zhang, Q., Shi, X., Wang, Q., Zhang, L., and Wang, X. (2020). Structure of the SARS-CoV-2 spike receptor-binding domain bound to the ACE2 receptor. Nature *581*, 215–220.

Lander, G.C., Stagg, S.M., Voss, N.R., Cheng, A., Fellmann, D., Pulokas, J., Yoshioka, C., Irving, C., Mulder, A., Lau, P.W., et al. (2009). Appion: an integrated, database-driven pipeline to facilitate EM image processing. J. Struct. Biol. *166*, 95–102.

CellPress
OPEN ACCESS

Lawrence, M.C., and Colman, P.M. (1993). Shape complementarity at protein/protein interfaces. J. Mol. Biol. *234*, 946–950.

Lee, P.S., Yoshida, R., Ekiert, D.C., Sakai, N., Suzuki, Y., Takada, A., and Wilson, I.A. (2012). Heterosubtypic antibody recognition of the influenza virus hemagglutinin receptor binding site enhanced by avidity. Proc. Natl. Acad. Sci. USA *109*, 17040–17045.

Letko, M., Marzi, A., and Munster, V. (2020). Functional assessment of cell entry and receptor usage for SARS-CoV-2 and other lineage B betacoronaviruses. Nat. Microbiol. *5*, 562–569.

Li, W., Drelich, A., Martinez, D.R., Gralinski, L., Chen, C., Sun, Z., Liu, X., Zhelev, D., Zhang, L., Peterson, E.C., et al. (2020). Potent neutralization of SARS-CoV-2 in vitro and in an animal model by a human monoclonal antibody. bioRxiv. https://doi.org/10.1101/2020.05.13.093088.

Liu, L., Wang, P., Nair, M.S., Yu, J., Rapp, M., Wang, Q., Luo, Y., Chan, J.F., Sahi, V., Figueroa, A., et al. (2020). Potent neutralizing antibodies against multiple epitopes on SARS-CoV-2 spike. Nature *584*, 450–456.

Lv, H., Wu, N.C., Tsang, O.T.-Y., Yuan, M., Perera, R.A.P.M., Leung, W.S., So, R.T.Y., Chan, J.M.C., Yip, G.K., Chik, T.S.H., et al. (2020). Cross-reactive antibody response between SARS-CoV-2 and SARS-CoV infections. Cell Rep. *31*, 107725.

McCoy, A.J., Grosse-Kunstleve, R.W., Adams, P.D., Winn, M.D., Storoni, L.C., and Read, R.J. (2007). Phaser crystallographic software. J. Appl. Cryst. *40*, 658–674.

McLellan, J.S., Pancera, M., Carrico, C., Gorman, J., Julien, J.P., Khayat, R., Louder, R., Pejchal, R., Sastry, M., Dai, K., et al. (2011). Structure of HIV-1 gp120 V1/V2 domain with broadly neutralizing antibody PG9. Nature *480*, 336–343.

Menachery, V.D., Yount, B.L., Jr., Debbink, K., Agnihothram, S., Gralinski, L.E., Plante, J.A., Graham, R.L., Scobey, T., Ge, X.Y., Donaldson, E.F., et al. (2015). A SARS-like cluster of circulating bat coronaviruses shows potential for human emergence. Nat. Med. *21*, 1508–1513.

Otwinowski, Z., and Minor, W. (1997). Processing of X-ray diffraction data collected in oscillation mode. Methods Enzymol. *276*, 307–326.

Pan, J., Peng, H., Chen, B., and Harrison, S.C. (2020). Cryo-EM structure of full-length HIV-1 Env bound with the Fab of antibody PG16. J. Mol. Biol. *432*, 1158–1168.

Pettersen, E.F., Goddard, T.D., Huang, C.C., Couch, G.S., Greenblatt, D.M., Meng, E.C., and Ferrin, T.E. (2004). UCSF Chimera–a visualization system for exploratory research and analysis. J. Comput. Chem. *25*, 1605–1612.

Pinto, D., Park, Y.J., Beltramello, M., Walls, A.C., Tortorici, M.A., Bianchi, S., Jaconi, S., Culap, K., Zatta, F., De Marco, A., et al. (2020). Cross-neutralization of SARS-CoV-2 by a human monoclonal SARS-CoV antibody. Nature *583*, 290–295.

Robbiani, D.F., Gaebler, C., Muecksch, F., Lorenzi, J.C.C., Wang, Z., Cho, A., Agudelo, M., Barnes, C.O., Gazumyan, A., Finkin, S., et al. (2020). Convergent antibody responses to SARS-CoV-2 in convalescent individuals. Nature *584*, 437–442.

Rogers, T.F., Zhao, F., Huang, D., Beutler, N., Burns, A., He, W.T., Limbo, O., Smith, C., Song, G., Woehl, J., et al. (2020). Isolation of potent SARS-CoV-2 neutralizing antibodies and protection from disease in a small animal model. Science *369*, 956–963.

Schmidt, F., Weisblum, Y., Muecksch, F., Hoffmann, H.-H., Michailidis, E., Lorenzi, J.C.C., Mendoza, P., Rutkowska, M., Bednarski, E., Gaebler, C., et al. (2020). Measuring SARS-CoV-2 neutralizing antibody activity using pseudotyped and chimeric viruses. J. Exp. Med. *217*, e20201181.

Seydoux, E., Homad, L.J., MacCamy, A.J., Parks, K.R., Hurlburt, N.K., Jennewein, M.F., Akins, N.R., Stuart, A.B., Wan, Y.-H., Feng, J., et al. (2020). Analysis of a SARS-CoV-2-infected individual reveals development of potent neutralizing antibodies with limited somatic mutation. Immunity *53*, 98–105.e5.

Shang, J., Wan, Y., Luo, C., Ye, G., Geng, Q., Auerbach, A., and Li, F. (2020). Cell entry mechanisms of SARS-CoV-2. Proc. Natl. Acad. Sci. USA *117*, 11727–11734.

Shi, R., Shan, C., Duan, X., Chen, Z., Liu, P., Song, J., Song, T., Bi, X., Han, C., Wu, L., et al. (2020). A human neutralizing antibody targets the receptor-binding site of SARS-CoV-2. Nature *584*, 120–124.

Strauch, E.M., Bernard, S.M., La, D., Bohn, A.J., Lee, P.S., Anderson, C.E., Nieusma, T., Holstein, C.A., Garcia, N.K., Hooper, K.A., et al. (2017). Computational design of trimeric influenza-neutralizing proteins targeting the hemagglutinin receptor binding site. Nat. Biotechnol. *35*, 667–671.

Suloway, C., Pulokas, J., Fellmann, D., Cheng, A., Guerra, F., Quispe, J., Stagg, S., Potter, C.S., and Carragher, B. (2005). Automated molecular microscopy: the new Leginon system. J. Struct. Biol. *151*, 41–60.

ter Meulen, J., van den Brink, E.N., Poon, L.L., Marissen, W.E., Leung, C.S., Cox, F., Cheung, C.Y., Bakker, A.Q., Bogaards, J.A., van Deventer, E., et al. (2006). Human monoclonal antibody combination against SARS coronavirus: synergy and coverage of escape mutants. PLoS Med. *3*, e237.

Tian, X., Li, C., Huang, A., Xia, S., Lu, S., Shi, Z., Lu, L., Jiang, S., Yang, Z., Wu, Y., and Ying, T. (2020). Potent binding of 2019 novel coronavirus spike protein by a SARS coronavirus-specific human monoclonal antibody. Emerg. Microbes Infect. *9*, 382–385.

Voss, N.R., Yoshioka, C.K., Radermacher, M., Potter, C.S., and Carragher, B. (2009). DoG Picker and TiltPicker: software tools to facilitate particle selection in single particle electron microscopy. J. Struct. Biol. *166*, 205–213.

Walls, A.C., Park, Y.J., Tortorici, M.A., Wall, A., McGuire, A.T., and Veesler, D. (2020). Structure, function, and antigenicity of the SARS-CoV-2 spike glycoprotein. Cell *181*, 281–292.e6.

Ward, A.B., and Wilson, I.A. (2020). Innovations in structure-based antigen design and immune monitoring for next generation vaccines. Curr. Opin. Immunol. *65*, 50–56.

Watanabe, A., McCarthy, K.R., Kuraoka, M., Schmidt, A.G., Adachi, Y., Onodera, T., Tonouchi, K., Caradonna, T.M., Bajic, G., Song, S., et al. (2019). Antibodies to a conserved influenza head interface epitope protect by an IgG subtype-dependent mechanism. Cell *177*, 1124–1135.e16.

Watanabe, Y., Allen, J.D., Wrapp, D., McLellan, J.S., and Crispin, M. (2020). Site-specific glycan analysis of the SARS-CoV-2 spike. Science *369*, 330–333.

Wec, A.Z., Wrapp, D., Herbert, A.S., Maurer, D.P., Haslwanter, D., Sakharkar, M., Jangra, R.K., Dieterle, M.E., Lilov, A., Huang, D., et al. (2020). Broad neutralization of SARS-related viruses by human monoclonal antibodies. Science *369*, 731–736.

Wrapp, D., De Vlieger, D., Corbett, K.S., Torres, G.M., Wang, N., Van Breedam, W., Roose, K., van Schie, L.; VIB-CMB COVID-19 Response Team, and Hoffmann, M., et al. (2020a). Structural basis for potent neutralization of betacoronaviruses by single-domain camelid antibodies. Cell *181*, 1004–1015.e15.

Wrapp, D., Wang, N., Corbett, K.S., Goldsmith, J.A., Hsieh, C.L., Abiona, O., Graham, B.S., and McLellan, J.S. (2020b). Cryo-EM structure of the 2019-nCoV spike in the prefusion conformation. Science *367*, 1260–1263.

Wu, N.C., and Wilson, I.A. (2018). Structural insights into the design of novel anti-influenza therapies. Nat. Struct. Mol. Biol. *25*, 115–121.

Wu, Y., Wang, F., Shen, C., Peng, W., Li, D., Zhao, C., Li, Z., Li, S., Bi, Y., Yang, Y., et al. (2020c). A noncompeting pair of human neutralizing antibodies block COVID-19 virus binding to its receptor ACE2. Science *368*, 1274–1278.

Wu, N.C., Yuan, M., Bangaru, S., Huang, D., Zhu, X., Lee, C.D., Turner, H.L., Peng, L., Yang, L., Nemazee, D., et al. (2020a). A natural mutation between SARS-CoV-2 and SARS-CoV determines neutralization by a cross-reactive antibody. bioRxiv. https://doi.org/10.1101/2020.09.21.305441.

Wu, N.C., Yuan, M., Liu, H., Lee, C.D., Zhu, X., Bangaru, S., Torres, J.L., Caniels, T.G., Brouwer, P.J.M., van Gils, M.J., et al. (2020b). An alternative binding mode of IGHV3-53 antibodies to the SARS-CoV-2 receptor binding domain. Cell Reports *33*, 108274.

Yan, R., Zhang, Y., Li, Y., Xia, L., Guo, Y., and Zhou, Q. (2020). Structural basis for the recognition of SARS-CoV-2 by full-length human ACE2. Science *367*, 1444–1448.

Yao, H., Song, Y., Chen, Y., Wu, N., Xu, J., Sun, C., Zhang, J., Weng, T., Zhang, Z., Wu, Z., et al. (2020). Molecular architecture of the SARS-CoV-2 virus. Cell *183*, 730–738.e13.

Yuan, M., Liu, H., Wu, N.C., Lee, C.D., Zhu, X., Zhao, F., Huang, D., Yu, W., Hua, Y., Tien, H., et al. (2020a). Structural basis of a shared antibody response to SARS-CoV-2. Science *369*, 1119–1123.

Yuan, M., Wu, N.C., Zhu, X., Lee, C.D., So, R.T.Y., Lv, H., Mok, C.K.P., and Wilson, I.A. (2020b). A highly conserved cryptic epitope in the receptor binding domains of SARS-CoV-2 and SARS-CoV. Science *368*, 630–633.

Yuan, M., Liu, H., Wu, N.C., and Wilson, I.A. (2020c). Recognition of the SARS-CoV-2 receptor binding domain by neutralizing antibodies. Biochem. Biophys. Res. Commun. Published online October 10, 2020. https://doi.org/10.1016/j.bbrc.2020.10.012.

Zhou, D., Duyvesteyn, H.M.E., Chen, C.-P., Huang, C.-G., Chen, T.-H., Shih, S.-R., Lin, Y.-C., Cheng, C.-Y., Cheng, S.-H., Huang, Y.-C., et al. (2020a). Structural basis for the neutralization of SARS-CoV-2 by an antibody from a convalescent patient. Nat. Struct. Mol. Biol. *27*, 950–958.

Zhou, P., Yang, X.L., Wang, X.G., Hu, B., Zhang, L., Zhang, W., Si, H.R., Zhu, Y., Li, B., Huang, C.L., et al. (2020b). A pneumonia outbreak associated with a new coronavirus of probable bat origin. Nature *579*, 270–273.

Zivanov, J., Nakane, T., Forsberg, B.O., Kimanius, D., Hagen, W.J., Lindahl, E., and Scheres, S.H. (2018). New tools for automated high-resolution cryo-EM structure determination in RELION-3. eLife *7*, e42166.

Zost, S.J., Gilchuk, P., Chen, R.E., Case, J.B., Reidy, J.X., Trivette, A., Nargi, R.S., Sutton, R.E., Suryadevara, N., Chen, E.C., et al. (2020). Rapid isolation and profiling of a diverse panel of human monoclonal antibodies targeting the SARS-CoV-2 spike protein. Nat. Med. *26*, 1422–1427.

STAR★METHODS

KEY RESOURCES TABLE

REAGENT or RESOURCE	SOURCE	IDENTIFIER
ExpiCHO Expression System Kit	Thermo Fisher Scientific	A29133
Expi293 Expression System Kit	Thermo Fisher Scientific	A14635
HyClone insect cell culture medium	GE Healthcare	SH30280.03
FreeStyle 293 expression medium	GIBCO	12338002
Opti-MEM I reduced serum media	GIBCO	51985091
Phosphate-buffered saline (PBS)	Thermo Fisher Scientific	14040133
Ni-NTA Superflow	QIAGEN	30450
DH10Bac competent cells	Thermo Fisher Scientific	10361012
CaptureSelect CH1-XL Affinity Matrix	Thermo Fisher Scientific	2943452010
Protein A column	Thermo Fisher Scientific	17040301
Chemicals and Recombinant Proteins		
DpnI	New England Biolabs	R0176L
Trypsin	New England Biolabs	P8101S
Fugene 6 Transfection Regent	Promega	E2691
BirA Biotin-Protein Ligase Reaction Kit	Avidity	BIRA-500
Sodium chloride (NaCl)	Sigma-Aldrich	S9888
Tris Base	Sigma-Aldrich	11814273001
Concentrated hydrochloric acid (HCl)	Sigma-Aldrich	H1758
Sodium azide (NaN_3)	Sigma-Aldrich	S2002
Bovine Serum Albumin (BSA)	Sigma-Aldrich	A9418
Tween 20	Fisher Scientific	BP337-500
PEImax	Polysciences	24765-1
Chemicals for protein crystallization	Hampton Research	N/A
Critical Commercial Assays		
In-Fusion HD Cloning Kit	Takara	639647
KOD Hot Start DNA Polymerase	EMD Millipore	71086-3
PCR Clean-Up and Gel Extraction Kit	Clontech Laboratories	740609.250
QIAprep Spin Miniprep Kit	QIAGEN	27106
NucleoBond Xtra Maxi	Clontech Laboratories	740414.100
Deposited Data		
X-ray coordinates and structure factors of COVA1-16 Fab	This study	PDB: 7JMX
X-ray coordinates and structure factors of COVA1-16 Fab in complex with SARS-CoV-2 RBD	This study	PDB: 7JMW
Cell Lines		
ExpiCHO cells	Thermo Fisher Scientific	A29127
Expi293F cells	Thermo Fisher Scientific	A14527
HEK293F cells	Invitrogen	R79007
Sf9 cells	ATCC	CRL-1711
High Five cells	Thermo Fisher Scientific	B85502
Recombinant DNA		
phCMV3-COVA1-16 IgG heavy chain	Brouwer et al., 2020	N/A
phCMV3-COVA1-16 Fab heavy chain	Brouwer et al., 2020	N/A
phCMV3-COVA1-16 light chain	Brouwer et al., 2020	N/A

(*Continued on next page*)

Continued

REAGENT or RESOURCE	SOURCE	IDENTIFIER
pPPI4-SARS-CoV RBD	Brouwer et al., 2020	N/A
pPPI4-SARS-CoV-2 RBD	Brouwer et al., 2020	N/A
pPPI4-SARS-CoV	Brouwer et al., 2020	N/A
pPPI4-SARS-CoV-2	Brouwer et al., 2020	N/A
pFastBac-SARS-CoV-RBD	Yuan et al., 2020b	N/A
pFastBac-SARS-CoV-2-RBD	Yuan et al., 2020b	N/A
phCMV3-ACE2	This study	N/A
Software and Algorithms		
HKL2000	Otwinowski and Minor, 1997	N/A
Phaser	McCoy et al., 2007	N/A
Coot	Emsley et al., 2010	N/A
Phenix	Adams et al., 2010	N/A
MolProbity	Chen et al., 2010	N/A
WebLogo	Crooks et al., 2004	N/A
PyMOL	Schrödinger, LLC	RRID:SCR_000305
Appion	Lander et al., 2009	N/A
DoG Picker	Voss et al., 2009	N/A
Relion	Zivanov et al., 2018	N/A
UCSF Chimera	Pettersen et al., 2004	N/A
Octet analysis software 9.0	Fortebio	https://www.moleculardevices.com/
Other		
Fab-CH1 2nd generation (FAB2G) biosensors	ForteBio	Cat# 18-5019
Ni-NTA biosensors	ForteBio	Cat# 18-5102
Streptavidin (SA) biosensors	ForteBio	Cat# 18-5020
Negative stain EM grids, 400 mesh	Electron Microscopy Sciences	EMS400-CU

RESOURCE AVAILABILITY

Lead Contact

Information and requests for resources and reagents should be directed to and will be fulfilled by the Lead Contact, Ian A. Wilson (wilson@scripps.edu).

Materials Availability

All reagents generated in this study are available from the Lead Contact (I.A.W.) with a completed Materials Transfer Agreement.

Data and Code Availability

X-ray coordinates and structure factors have been deposited in the RCSB Protein Data Bank under accession codes PDB: 7JMW and 7JMX. COVA1-16 IGVH and IGVK sequences are available in GenBank: MT599835 and MT599919. The EM maps have been deposited at the Electron Microscopy Data Bank (EMDB) with accession codes EMDB: EMD-22742, EMD-22743, EMD-22744, and EMD-22745.

EXPERIMENTAL MODEL AND SUBJECT DETAILS

Expression and Purification of SARS-CoV, SARS-CoV-2, and SARSr-CoV RBDs

The receptor-binding domain (RBD) (residues 319-541) of the SARS-CoV-2 spike (S) protein (GenBank: QHD43416.1), the RBD (residues 306-527) of the SARS-CoV S protein (GenBank: ABF65836.1), the RBD (residues 315-537) of pangolin-CoV (GenBank: QLR06866.1), and the RBD (residues 319-541) of Bat-CoV RaTG13 (GenBank: QHR63300.2) were cloned into a customized pFast-Bac vector (Ekiert et al., 2011), and fused with an N-terminal gp67 signal peptide and C-terminal His_6 tag (Yuan et al., 2020b). For each RBD, we further cloned a construct with an AviTag inserted in front of the His_6 tag. To express the RBD, a recombinant bacmid DNA was generated using the Bac-to-Bac system (Life Technologies). Baculovirus was generated by transfecting purified bacmid DNA into Sf9 cells using FuGENE HD (Promega), and subsequently used to infect suspension cultures of High Five cells (Life Technologies)

at an MOI of 5 to 10. Infected High Five cells were incubated at 28 °C with shaking at 110 rpm for 72 h for protein expression. The supernatant was then concentrated using a 10 kDa MW cutoff Centramate cassette (Pall Corporation). The RBD protein was purified by Ni-NTA, followed by size exclusion chromatography, and buffer exchanged into 20 mM Tris-HCl pH 7.4 and 150 mM NaCl. For binding experiments, RBD with AviTag was biotinylated as described previously (Ekiert et al., 2012) and purified by size exclusion chromatography on a Hiload 16/90 Superdex 200 column (GE Healthcare) in 20 mM Tris-HCl pH 7.4 and 150 mM NaCl.

Expression and Purification of COVA1-16 Fab

Expression plasmids encoding the heavy and light chains of the COVA1-16 Fab were transiently co-transfected into ExpiCHO cells at a ratio of 2:1 (HC:LC) using ExpiFectamine CHO Reagent (Thermo Fisher Scientific) according to the manufacturer's instructions. The supernatant was collected at 10 days post-transfection. The Fabs were purified with a CaptureSelect CH1-XL Affinity Matrix (Thermo Fisher Scientific) followed by size exclusion chromatography.

Expression and Purification of ACE2

The N-terminal peptidase domain of human ACE2 (residues 19 to 615, GenBank: BAB40370.1) was cloned into phCMV3 vector and fused with a C-terminal Fc tag. The plasmids were transiently transfected into Expi293F cells using ExpiFectamine 293 Reagent (Thermo Fisher Scientific) according to the manufacturer's instructions. The supernatant was collected at 7 days post-transfection. Fc-tagged ACE2 protein was then purified with a Protein A column (GE Healthcare) followed by size exclusion chromatography.

Crystallization and X-ray Structure Determination

The COVA1-16 Fab complex with RBD was formed by mixing each of the protein components in an equimolar ratio and incubating overnight at 4°C. The COVA1-16 Fab–RBD complex and COVA1-16 Fab apo (unliganded) protein were adjusted to around 11 mg/mL and screened for crystallization using the 384 conditions of the JCSG Core Suite (QIAGEN) on our custom-designed robotic CrystalMation system (Rigaku) at Scripps Research. Crystallization trials were set-up by the vapor diffusion method in sitting drops containing 0.1 μL of protein and 0.1 μL of reservoir solution. Crystals used for X-ray data collection were harvested from drops containing 0.2 M sodium iodide and 20% (w/v) polyethylene glycol 3350 for the COVA1-16 Fab–RBD complex and from drops containing 0.08 M acetate pH 4.6, 20% (w/v) polyethylene glycol 4000, 0.16 M ammonium sulfate and 20% (v/v) glycerol for the COVA1-16 Fab. Crystals appeared on day 3, were harvested on day 7, pre-equilibrated in cryoprotectant containing 20% glycerol, and then flash cooled and stored in liquid nitrogen until data collection. Diffraction data were collected at cryogenic temperature (100 K) at Stanford Synchrotron Radiation Lightsource (SSRL) on the Scripps/Stanford beamline 12-1 with a beam wavelength of 0.97946 Å, and processed with HKL2000 (Otwinowski and Minor, 1997). Structures were solved by molecular replacement using PHASER (McCoy et al., 2007). The models for molecular replacement of RBD and COVA1-16 were from PDB: 6XC4 (Yuan et al., 2020a), 4IMK (Fenn et al., 2013) and 2Q20 (Baden et al., 2008). Iterative model building and refinement were carried out in COOT (Emsley et al., 2010) and PHENIX (Adams et al., 2010), respectively. Ramachandran statistics were calculated by MolProbity (Chen et al., 2010). Epitope and paratope residues, as well as their interactions, were identified by accessing PISA software server (https://www.ebi.ac.uk/pdbe/prot_int/pistart.html; Krissinel and Henrick, 2007).

Expression and Purification of Recombinant S Protein for Negative-Stain EM

The SARS-CoV-2 S construct used for negative-stain EM contains the mammalian-codon-optimized gene encoding residues 1-1208 of the S protein (GenBank: QHD43416.1), followed by a C-terminal T4 fibritin trimerization domain, an HRV3C cleavage site, 8x-His tag and a Twin-strep tags subcloned into the eukaryotic-expression vector pcDNA3.4. Three amino-acid mutations were introduced into the S1–S2 cleavage site (RRAR to GSAS) to prevent cleavage and two stabilizing proline mutations (K986P and V987P) to the HR1 domain. For additional S stabilization, residues T883 and V705 were mutated to cysteines to introduce a disulphide bond. The S plasmid was transfected into 293F cells and supernatant was harvested at 6 days post transfection. S protein was purified by running the supernatant through a streptactin column and then by size exclusion chromatography using a Superose 6 increase 10/300 column (GE Healthcare Biosciences). Protein fractions corresponding to the trimeric S protein were collected and concentrated.

nsEM Sample Preparation and Data Collection

SARS-CoV-2 S protein was complexed with 3x molar excess of Fab for 30 min prior to direct deposition onto carbon-coated 400-mesh copper grids. The grids were stained with 2% (w/v) uranyl-formate for 90 s immediately following sample application. Grids were either imaged at 200 keV or at 120 keV on a Tecnai T12 Spirit using a 4kx4k Eagle CCD. Micrographs were collected using Leginon (Suloway et al., 2005) and the images were transferred to Appion for processing. Particle stacks were generated in Appion (Lander et al., 2009) with particles picked using a difference-of-Gaussians picker (DoG-picker) (Voss et al., 2009). Particle stacks were then transferred to Relion (Zivanov et al., 2018) for 2D classification followed by 3D classification to sort well-behaved classes. Selected 3D classes were auto-refined on Relion and used to make figures with UCSF Chimera (Pettersen et al., 2004). A published prefusion spike model (PDB: 6Z97) (Huo et al., 2020) was used in our structural analysis.

Protein Expression and Purification for Antibody Binding Studies

All constructs were expressed transiently in HEK293F (Invitrogen, cat no. R79009) cells maintained in Freestyle medium (Life Technologies). For soluble RBD proteins, cells were transfected at a density of 0.8-1.2 million cells/mL by addition of a mix of PEImax (1 μg/μL) with expression plasmids (312.5 μg/L) in a 3:1 ratio in OptiMEM. Supernatants of the soluble RBD proteins were harvested six days post transfection, centrifuged for 30 min at 4000 rpm and filtered using 0.22 μm Steritop filters (Merck Millipore). Constructs with a His_6-tag were purified by affinity purification using Ni-NTA agarose beads. Protein eluates were concentrated, and buffer exchanged to PBS using Vivaspin filters with a 10 kDa molecular weight cutoff (GE Healthcare). Protein concentrations were determined by Nanodrop using the proteins peptidic molecular weight and extinction coefficient as determined by the online ExPASy software (ProtParam). For the COVA1-16 IgG1 antibody, suspension HEK293F cells (Invitrogen, cat no. R79007) were cultured in FreeStyle medium (GIBCO) and co-transfected with the two IgG plasmids expressing the corresponding HC and LC in a 1:1 ratio at a density of 0.8-1.2 million cells/mL in a 1:3 ratio with 1 mg/L PEImax (Polysciences). The recombinant IgG antibodies were isolated from the cell supernatant after five days as described previously (20, 48). In short, the cell suspension was centrifuged 25 min at 4000 rpm, and the supernatant was filtered using 0.22 μm pore size SteriTop filters (Millipore). The filtered supernatant was run over a 10 mL protein A/G column (Pierce) followed by two column volumes of PBS wash. The antibodies were eluted with 0.1 M glycine pH 2.5, into the neutralization buffer of 1 M TRIS pH 8.7 in a 1:9 ratio. The purified antibodies were buffer exchanged to PBS using 100 kDa VivaSpin20 columns (Sartorius). The IgG concentration was determined on the NanoDrop 2000 and the antibodies were stored at 4°C until further analyses.

Measurement of Binding Affinities Using Biolayer Interferometry

To determine the binding affinity of COVA1-16 IgG and His-tagged Fabs, 20 μg/mL of His-tagged SARS-CoV or SARS-CoV-2 RBD protein in running buffer (PBS, 0.02% Tween-20, 0.1% BSA) was loaded on Ni-NTA biosensors (ForteBio) for 300 s. Streptavidin biosensors (ForteBio) were used if the RBD was biotinylated. Next, the biosensors were transferred to running buffer containing IgG or Fab to determine the association rate, after which the sensor was transferred to a well containing running buffer to allow dissociation. As negative control, an anti-HIV-1 His-tagged Fab was tested at the highest concentration used for COVA1-16 Fab (400 nM). After each cycle, the sensors were regenerated by alternating 20 mM glycine in PBS and running buffer three times, followed by reactivation in 20 mM $NiCl_2$ for 120 s. All steps were performed at 1000 rpm shaking speed. K_Ds were determined using ForteBio Octet CFR software. The avidity effects of IgG were investigated by titrating the SARS-CoV-2 RBD concentration (5, 1, 0.2 and 0.04 μg/mL) followed by loading on Ni-NTA biosensors for 480 s with an additional loading step with His-tagged HIV-1 gp41 for 480 s to minimize background binding of His-tagged Fabs to the biosensor. All other steps were performed as described above.

Competition Studies of Antibodies with ACE2 Receptor

For competition assays, COVA1-16 IgG, CR3022 IgG, and human ACE2-Fc were all diluted to 250 nM. Ni-NTA biosensors were used. In brief, the assay has five steps: 1) baseline: 60 s with 1x kinetics buffer; 2) loading: 180 s with 20 μg/mL, His_6-tagged SARS-CoV-2 RBD proteins; 3) baseline: 150 s with 1x kinetics buffer; 4) first association: 300 s with CR3022 IgG or human ACE2-Fc; and 5) second association: 300 s with human ACE2-Fc, CR3022 IgG, or COVA1-16 IgG.

Pseudovirus Neutralization Assay

Neutralization assays were performed using SARS-CoV and SARS-CoV-2 S-pseudotyped HIV-1 virus and HEK293T–ACE2 cells as described previously (Schmidt et al., 2020). In brief, pseudotyped virus was produced by co-transfecting expression plasmids of SARS-CoV S and SARS-CoV-$2_{\Delta 19}$ S proteins (GenBank; AAP33697.1 and MT449663.1, respectively) with an HIV backbone expressing NanoLuc luciferase (pHIV-1_{NL4-3} ΔEnv-NanoLuc) in HEK293T cells (ATCC, CRL-11268). After 3 days, the cell culture supernatants containing SARS-CoV and SARS-CoV-2 S-pseudotyped HIV-1 viruses were stored at −80°C. HEK293T–ACE2 cells were seeded 10,000 cells/well in a 96-well plate one day prior to the start of the neutralization assay. To determine the neutralizing capacity of COVA1-16 IgG and His_6-tagged Fab, 20 or 100 μg/mL COVA1-16 IgG and equal molar of COVA1-16 Fab were serially diluted in 3-fold steps and mixed with SARS-CoV or SARS-CoV-2 pseudotyped virus and incubated for 1 h at 37°C. The pseudotyped virus and COVA1-16 IgG or Fab mix were then added to the HEK293T–ACE2 cells and incubated at 37°C. After 48 h, cells were washed twice with PBS (Dulbecco's Phosphate-Buffered Saline, eBiosciences) and lysis buffer was added. Luciferase activity of cell lysate was measured using the Nano-Glo Luciferase Assay System (Promega) and GloMax Discover System. The inhibitory concentration (IC_{50}) was determined as the concentration of IgG or Fab that neutralized 50% of the pseudotyped virus using GraphPad Prism software (version 8.3.0).

Shape Complementarity Analysis

Shape complementarity values (Sc) were calculated as described by Lawrence and Colman (1993).

Sequence Conservation Analysis

RBD protein sequences from SARS-CoV and SARS-related coronavirus (SARSr-CoV) strains were retrieved from the following accession codes:

- GenBank ABF65836.1 (SARS-CoV)

- GenBank ALK02457.1 (Bat SARSr-CoV WIV16)
- GenBank AGZ48828.1 (Bat SARSr-CoV WIV1)
- GenBank ACU31032.1 (Bat SARSr-CoV Rs672)
- GenBank AIA62320.1 (Bat SARSr-CoV GX2013)
- GenBank AAZ67052.1 (Bat SARSr-CoV Rp3)
- GenBank AIA62300.1 (Bat SARSr-CoV SX2013)
- GenBank ABD75323.1 (Bat SARSr-CoV Rf1)
- GenBank AIA62310.1 (Bat SARSr-CoV HuB2013)
- GenBank AAY88866.1 (Bat SARSr-CoV HKU3-1)
- GenBank AID16716.1 (Bat SARSr-CoV Longquan-140)
- GenBank AVP78031.1 (Bat SARSr-CoV ZC45)
- GenBank AVP78042.1 (Bat SARSr-CoV ZXC21)
- GenBank QHR63300.2 (Bat CoV RaTG13)
- NCBI Reference Sequence YP_003858584.1 (Bat SARSr-CoV BM48-31)
- GISAID EPI_ISL_410721 (Pangolin BetaCoV Guandong2019)

Multiple sequence alignment of the RBD sequences was performed by MUSCLE version 3.8.31 (Edgar, 2004). Sequence logos were generated by WebLogo (Crooks et al., 2004). The conservation score of each RBD residue was calculated and mapped onto the SARS-CoV-2 RBD X-ray structure with ConSurf (Ashkenazy et al., 2016).

QUANTIFICATION AND STATISTICAL ANALYSIS

Statistical analysis was not performed in this study.

Immunity, Volume *53*

Supplemental Information

Cross-Neutralization of a SARS-CoV-2 Antibody to a Functionally Conserved Site Is Mediated by Avidity

Hejun Liu, Nicholas C. Wu, Meng Yuan, Sandhya Bangaru, Jonathan L. Torres, Tom G. Caniels, Jelle van Schooten, Xueyong Zhu, Chang-Chun D. Lee, Philip J.M. Brouwer, Marit J. van Gils, Rogier W. Sanders, Andrew B. Ward, and Ian A. Wilson

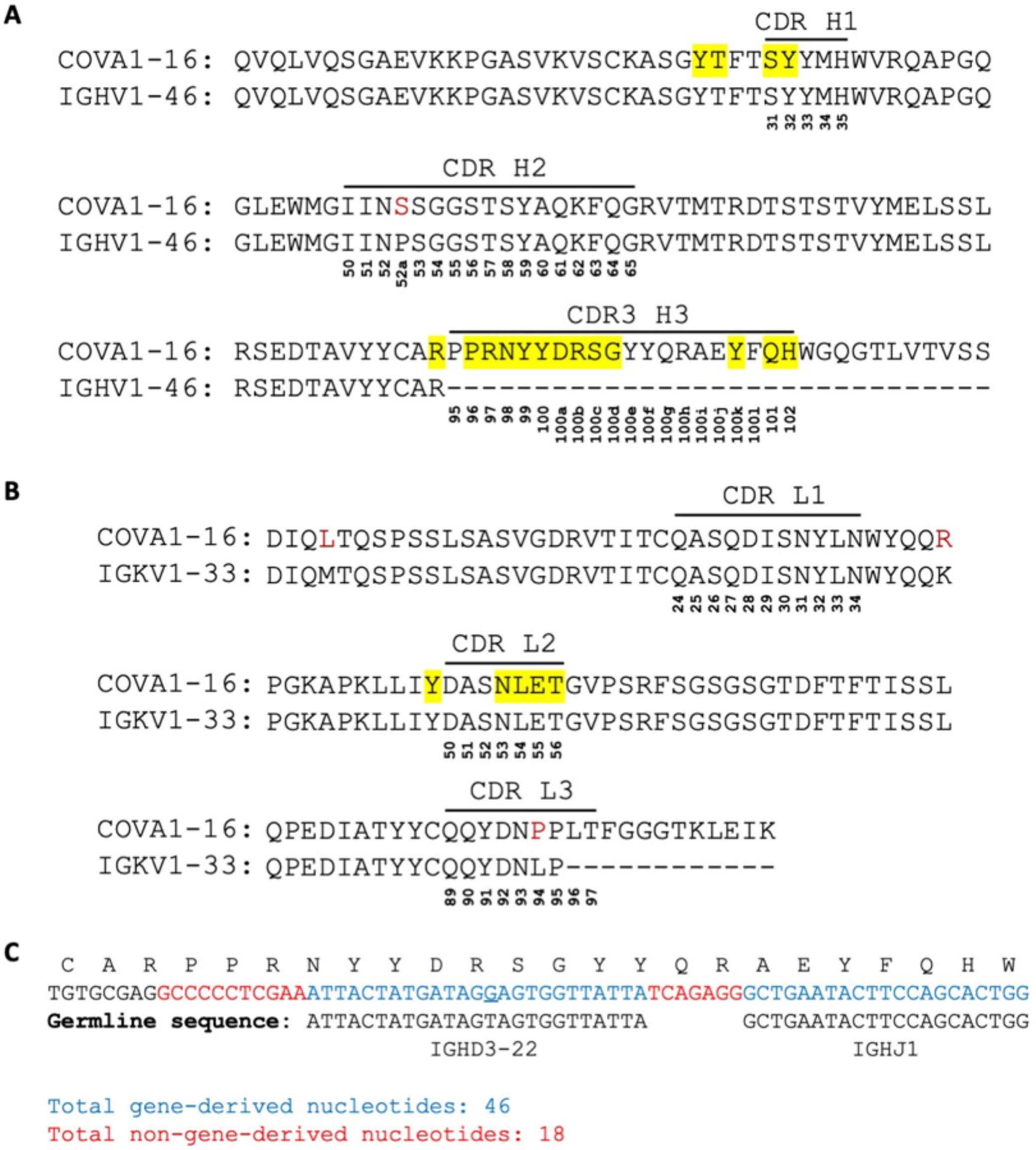

Figure S1, related to Figure 1. Comparison of COVA1-16 and putative germline sequences. Alignment of COVA1-16 Fab amino-acid sequence with **(A)** germline IGHV1-46 sequence, and **(B)** germline IGKV1-33 sequence. The regions that correspond to CDR H1, H2, H3, L1, L2, and L3 are indicated. Residues that differ from germline are highlighted in red. COVA1-16 Fab residues that interact with the RBD are highlighted in yellow [defined here as residues with a BSA > 0 Å^2 as calculated by the PISA program (Krissinel and Henrick, 2007)]. Residue positions in the CDRs are labeled according to the Kabat

numbering scheme. **(C)** Amino acid and nucleotide sequences of the V-D-J junction of COVA1-16, with putative gene segments (blue) and N-regions from N-addition (red), are indicated. The germline sequences of IGHD3-22 and IGHJ1 are also shown. The only somatically mutated nucleotide in the D region is underlined that results in a V_H S100bR mutation.

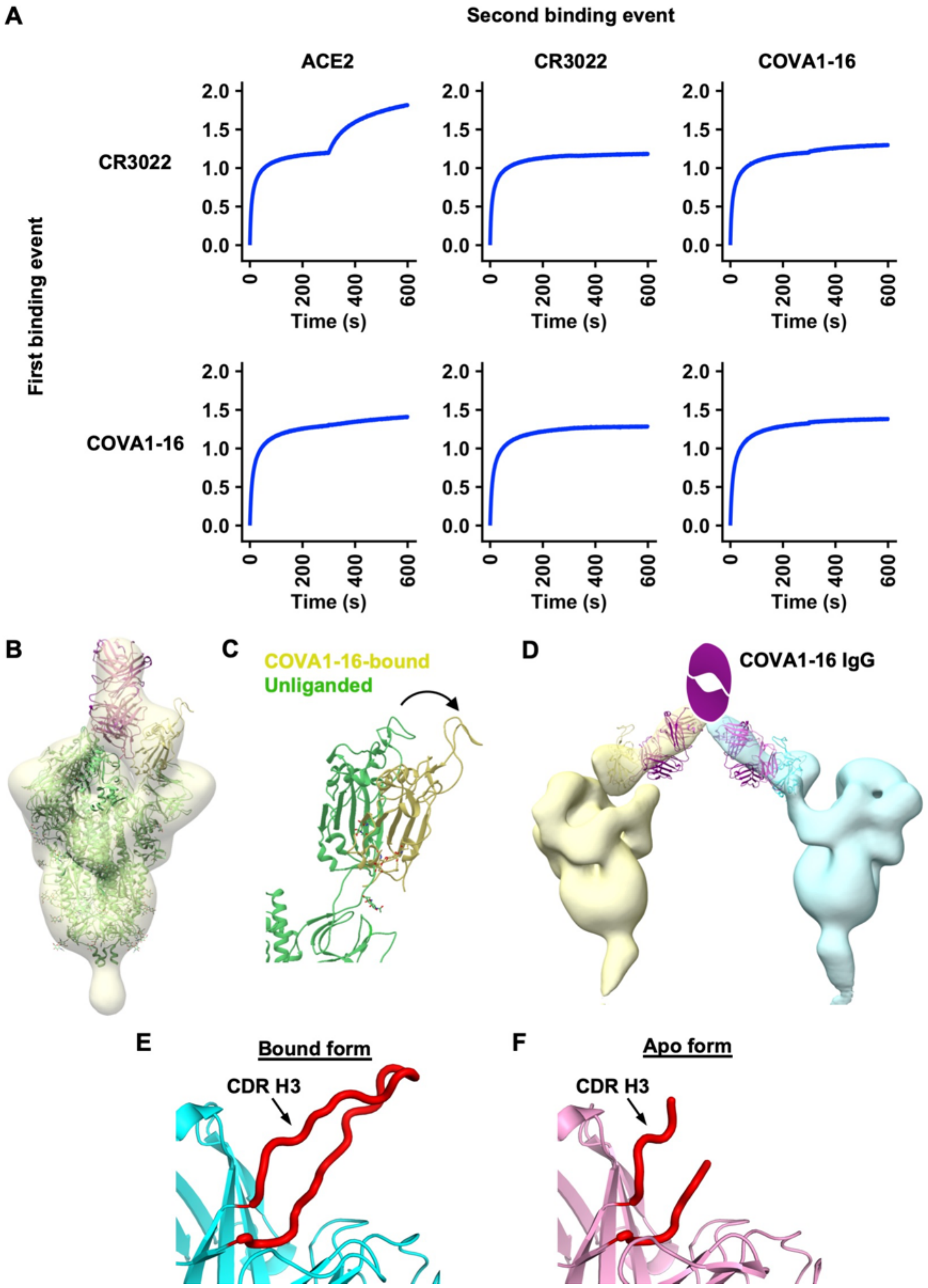

Figure S2, related to Figures 2 and 3. Competition assay between different IgGs and ACE2 and negative-stain EM analysis of COVA1-16 binding to SARS-CoV-2 S trimer.

Competition between COVA1-16 IgG, CR3022 IgG, and Fc-tagged ACE2 was measured by biolayer interferometry (BLI). Y-axis represents the response. The biosensor was first loaded with SARS-CoV-2 RBD, followed by two binding events: 1) CR3022 IgG or COVA1-16 IgG, and 2) ACE2, CR3022 IgG, or COVA1-16 IgG. A period of 300 s was used for each binding event. A further increase in signal during the second binding event (starting at 300 s time point) indicates lack of competition with the first ligand. **(B)** An atomic model from the crystal structure of SARS-CoV-2 RBD bound to COVA1-16 Fab was fit into the negative-stain EM reconstruction of the SARS-CoV-2 spike bound to COVA1-16 Fab. The COVA1-16 Fab approaches the apex of the S trimer in a perpendicular orientation. A secondary structure backbone representation of the prefusion spike model (PDB: 6Z97, green) (Huo et al., 2020) was also fit into the EM density with RBD residues (334-528) removed from one of the protomers here for clarity. The COVA1-16 heavy and light chains are in magenta and pink, respectively, and COVA1-16-bound RBD in yellow. **(C)** Conformation of RBD in an up conformation from an unliganded SARS-CoV-2 S trimer (PDB: 6Z97, green) (Huo et al., 2020) is compared to that of the RBD (yellow) bound by COVA1-16 Fab. The arrow indicates that the RBD further rotates and opens up when bound to COVA1-16, thereby moving further away from the trimer threefold axis. **(D)** An atomic model of the spike RBD bound to COVA1-16 Fab is fit into a negative-stain EM reconstruction, where COVA1-16 Fab approaches the SARS-CoV-2 S trimer from the side. COVA1-16 is modelled as an IgG to illustrate the feasibility of bivalent binding to adjacent spike proteins on the virus surface. The Fab heavy and light chains are shown in magenta and pink. A schematic representation of the Fc domain of the IgG is shown in magenta. The RBD model and spike density for each trimer is shown in yellow and cyan. **(E)** In the crystal structure of the RBD-bound form of COVA1-16 Fab, the CDR H3 loop is completely ordered (red). **(F)** In the crystal structure of the apo form of COVA1-16, the distal end of the CDR H3 loop is intrinsically disordered or flexible (red).

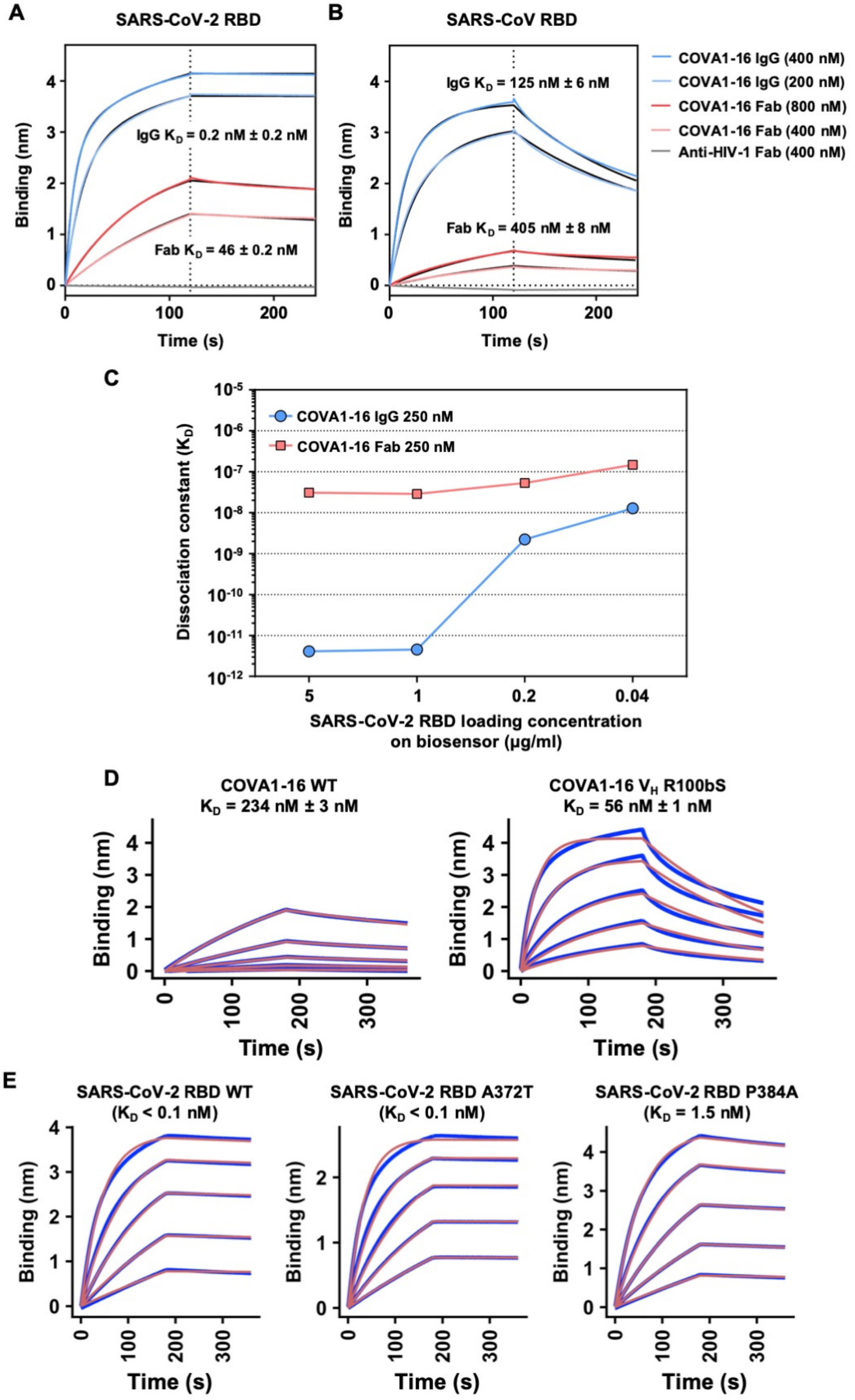

Figure S3, related to Figures 2 and 3. Sensorgrams for binding of COVA1-16 to SARS-CoV-2 RBD and SARS-CoV RBD. (A-B) Binding kinetics of COVA1-16 Fab and

IgG to **(A)** SARS-CoV-2 RBD and **(B)** SARS-CoV RBD were measured by biolayer interferometry (BLI) with RBD on the biosensor and antibody in solution. An anti-HIV His-tagged Fab (4E1) was used as a negative control. **(C)** The relationship between SARS-CoV-2 RBD loading concentration on the biosensor and the dissociation constant of COVA1-16 IgG is shown. **(D)** Binding kinetics of COVA1-16 wild-type and V_H R100bS mutant Fab to SARS-CoV-2 RBD were measured by biolayer interferometry (BLI) with RBD on the biosensor and antibody in solution. Unlike panels **A-C**, which used HEK293F-expressed SARS-CoV-2, the experiment here used insect cell-expressed SARS-CoV-2. **(E)** Binding kinetics of COVA1-16 IgG to SARS-CoV-2 RBD WT, A372T, and P384A were measured by biolayer interferometry (BLI) with RBD on the biosensor and antibody in solution. A372T and P384A are the only two mutations that differ between the SARS-CoV-2 and SARS-CoV sequences in COVA1-16 epitope. The affinity of COVA1-16 IgG to the A372T mutant did not show any detectable difference from WT. Although the affinity (K_D) of COVA1-16 IgG to the P384A mutant decreases, the binding is still 100 times tighter than that measured between COVA1-16 IgG and SARS-CoV RBD (see panel **B**). For all sensorgrams in this figure, Y-axis represents the response. Dissociation constants (K_D) for IgG and Fab were obtained using a 1:2 bivalent model and 1:1 binding model, respectively, which are represented by the red lines. Representative results of two replicates for each experiment are shown.

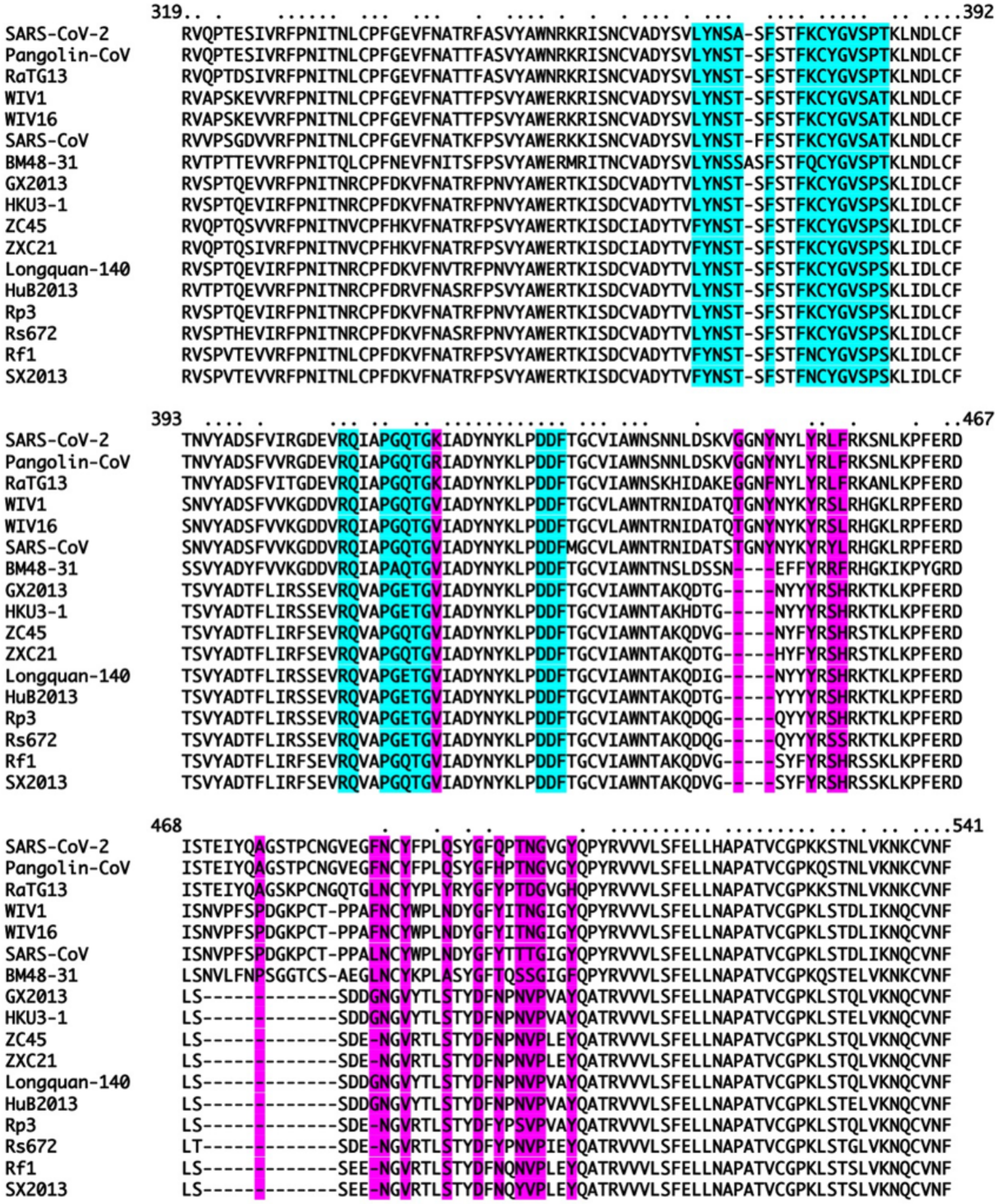

Figure S4, related to Figure 4. Sequence alignment of the RBD from SARS-related coronaviruses. Amino-acid sequences of RBDs from SARS-CoV-2, SARS-CoV, and other SARS-related coronavirus (SARSr-CoV) strains are aligned. COVA1-16 epitope residues are highlighted in cyan. ACE2-binding residues are highlighted in purple. Conserved residues are indicated by small black dots on the top of the alignment.

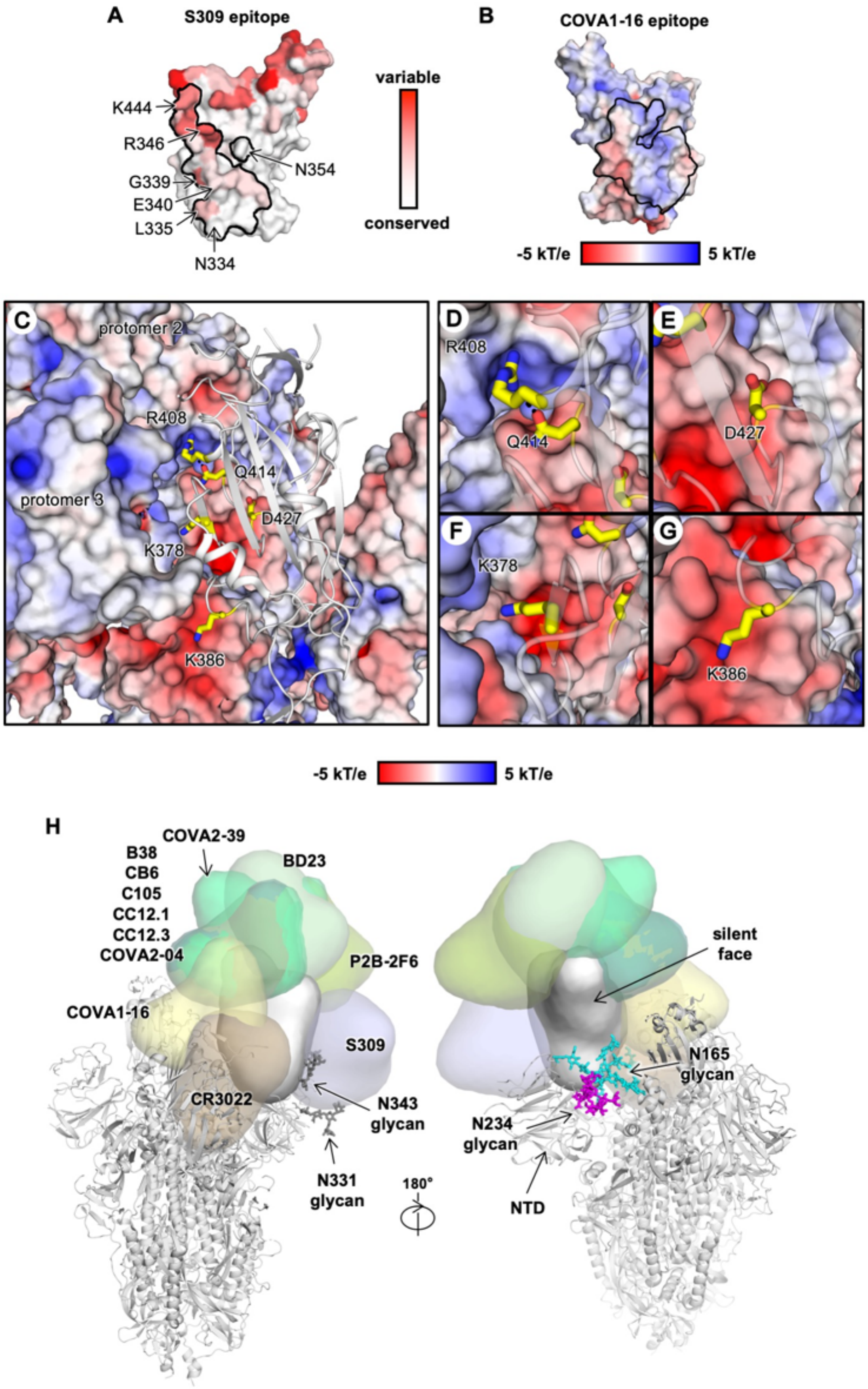

Figure S5, related to Figures 4 and 5. Sequence conservation of S309 epitope and additional structural analyses on COVA1-16 epitope. (A) Sequence conservation of

the RBD is highlighted on the structure for S309 epitope (Pinto et al., 2020). This view corresponds to the opposite side (rotated 180 degrees along the vertical axis) from that shown in Figure 4A-B. **(B)** The epitope of COVA1-16 is outlined and is mainly polar in character. **(C)** The RBD of one of the three protomers is shown as a gray cartoon with the side chains of five residues of interest shown in yellow stick representation. RBD residues K378, R408, Q414, and D427 are within the COVA1-16 epitope, whereas K386 is not a COVA1-16 epitope residue. The other two protomers (protomers 2 and 3) are shown in a surface electrostatic representation. **(D-G)** Zoomed-in views for the regions surrounding residues **(D)** R408 and Q414, **(E)** D427, **(F)** K378, and **(G)** K386**.** A hydrogen bond in **(D)** is represented by a dashed line. Due to charge difference or similarity between the side chain and the proximal region of the neighboring protomer, either repulsive (same charge) or attractive (opposite charge) environments are found and visualized here. PDB 6VXX is used to represent the spike protein (Walls et al., 2020). Of note, the shape complementarity values (Sc) (Lawrence and Colman, 1993) of the COVA1-16 epitope/RBD interface, COVA1-16 epitope/S2 interface, and COVA1-16 epitope/COVA1-16 interface are 0.53, 0.75, and 0.74, respectively, indicating good complementarity and tight fit of the COVA1-16 epitope surface with the rest of the trimer in the RBD down conformation. Sc values can range from 0 to 1, with a larger Sc value represents higher shape complementarity. **(H)** The antibody-bound RBD is shown in the up conformation on the S protein (PDB 6VSB) (Wrapp et al., 2020). N-glycans on N165 (NTD), N234, N331, and N343 (RBD) are modelled according to the main glycoform observed at these sites in (Watanabe et al., 2020) and shown in stick representation. Antibody Fabs from published crystal and cryo-EM structures are represented as globular outlines in different colors. B38, CB6, C105, CC12.1, CC12.3, COVA2-04, COVA2-39, BD23, P2B-2F6 all bind at or around the receptor binding site. S309 binds to the elongated accessible face of the RBD

in both up and down conformations, and CR3022 binds to the opposite face that is exposed in the RBD up conformation, but buried in the RBD down conformation.

Table S1, related to Figure 1. X-ray data collection and refinement statistics

**Data collection**		
	COVA1-16 Fab + SARS-CoV-2 RBD	COVA1-16 Fab
Beamline	SSRL 12-1	SSRL 12-1
Wavelength (Å)	0.97946	0.97946
Space group	P 1 2_1 1	P 4_1 3 2
Unit cell parameters		
a, b, c (Å)	57.4, 124.9, 57.6	156.3, 156.3, 156.3
α, β, γ (°)	90, 96.1, 90	90, 90, 90
Resolution (Å) [a]	50.0-2.89 (2.95-2.89)	50.0-2.53 (2.58-2.53)
Unique reflections [a]	17,656 (845)	22,357 (1,084)
Redundancy [a]	3.7 (3.2)	37.0 (14.1)
Completeness (%) [a]	97.9 (93.9)	100.0 (100.0)
$<I/\sigma_I>$ [a]	7.4 (1.2)	21.5 (1.3)
R_{sym}[b] (%) [a]	15.3 (69.1)	23.6 (>100)
R_{pim}[b] (%) [a]	9.0 (42.9)	3.8 (54.3)
$CC_{1/2}$[c] (%) [a]	96.3 (66.8)	99.6 (52.1)
**Refinement statistics**		
Resolution (Å)	42.8-2.89	34.1-2.53
Reflections (work)	17,632	21,872
Reflections (test)	948	1,069
R_{cryst}[d] / R_{free}[e] (%)	23.7/29.4	21.2/24.4
No. of atoms	4,873	3,284
Macromolecules	4,845	3,223
Glycans	28	-
Average *B*-values (Å^2)	49	43
Macromolecules	49	43
Fab	45	43
RBD	56	-
Glycans	89	-
Wilson *B*-value (Å^2)	43	40
**RMSD from ideal geometry**		
Bond length (Å)	0.004	0.007
Bond angle (°)	0.74	1.02
**Ramachandran statistics (%)** [f]		
Favored	95.9	96.7
Outliers	0.16	0.0
**PDB code**	7JMW	7JMX

[a] Numbers in parentheses refer to the highest resolution shell.

[b] $R_{sym} = \Sigma_{hkl} \Sigma_i | I_{hkl,i} - <I_{hkl}> | / \Sigma_{hkl} \Sigma_i I_{hkl,i}$ and $R_{pim} = \Sigma_{hkl} (1/(n-1))^{1/2} \Sigma_i | I_{hkl,i} - <I_{hkl}> | / \Sigma_{hkl} \Sigma_i I_{hkl,i}$, where $I_{hkl,i}$ is the scaled intensity of the ith measurement of reflection h, k, l, $<I_{hkl}>$ is the average intensity for that reflection, and n is the redundancy.

[c] $CC_{1/2}$ = Pearson correlation coefficient between two random half datasets.

[d] $R_{cryst} = \Sigma_{hkl} | F_o - F_c | / \Sigma_{hkl} | F_o |$ x 100, where F_o and F_c are the observed and calculated structure factors, respectively.

[e] R_{free} was calculated as for R_{cryst}, but on a test set comprising 5% of the data excluded from refinement.

[f] From MolProbity (Chen et al., 2010).

Table S2, related to Figure 1. Hydrogen bonds identified in the antibody-RBD interface using the PISA program

COVA1-16 Fab	Distance [Å]	SARS-CoV-2 RBD
H:ARG100b[NH2]	3.3	A:TYR369[O]
H:ARG100b[NE]	3.9	A:SER371[O]
H:ARG100b[N]	3.8	A:PHE377[O]
H:TYR100[N]	2.6	A:CYS379[O]
H:GLN101[NE2]	3.1	A:GLN414[OE1]
H:ARG97[NH1]	2.5	A:ASP427[O]
H:TYR32[OH]	3.1	A:ASP427[OD1]
H:THR28[N]	3.2	A:ASP427[OD2]
H:ARG97[NH1]	3.0	A:PHE429[O]
H:TYR100[O]	2.9	A:CYS379[N]
H:SER100c[O]	3.3	A:THR385[OG1]
H:GLN101[OE1]	3.8	A:GLN414[NE2]
L:ASN53[OD1]	3.2	A:ARG408[NH2]
L:LEU54[O]	3.7	A:ARG408[NE]
